# Genetically modified food and consumer risk responsibility: The effect of regulatory design and risk type on cognitive information processing

**DOI:** 10.1371/journal.pone.0252580

**Published:** 2021-06-09

**Authors:** Ashkan Pakseresht, Anna Kristina Edenbrandt, Carl Johan Lagerkvist

**Affiliations:** Department of Economics, Swedish University of Agricultural Sciences, Uppsala, Sweden; Universiteit Gent, BELGIUM

## Abstract

The use of agro-biotechnology has raised consumer concerns about environmental, health, socio-economic and ethical risks. This study examines how regulatory policies regarding genetically modified (GM) food production affect consumers’ cognitive information processing, in terms of perceived risk, self-control, and risk responsibility. There is further analysis of whether the effect of policy design is moderated by risk type. Data was generated in a field experiment (n = 547), including four different policy scenario treatments (banned, research and development, import, and full commercialization). The results reveal that policy scenarios where GM food is available on the market are associated with higher levels of perceived risk and lower levels of self-control compared with policies where GM food is banned. There was no evidence of policy scenarios affecting consumer willingness to assign personal risk responsibility. However, among participants who indicated health risks as their main concern, there was an effect from the policy scenario on self-risk responsibility as mediated through perceived risk and self-control. The results suggest that health-conscious consumers tend to attribute less responsibility to themselves in situations where a genetically modified product was commercialized. These findings indicate a need to clarify guideline recommendations for health-related risks associated with foods derived from biotechnology.

## 1. Introduction

The application of biotechnology in food production has been a contentious issue in Europe and indeed a topic of worldwide controversy in the past few decades. The European Commission recently approved new rules (Directive 2015/412) with provisions for opt-outs for member states from the Europe-wide approval system for food derived from biotechnology. Member states are permitted to institute an unlimited, or case-specific, moratorium on the commercial release of GM food within their respective territories and localities. These changes in EU regulations have the potential to affect the perception of associated risks among members of the public [1] and have consequences for consumers. Further, while products that contain GM (imported or domestically produced) must be labeled as such in the EU market, it has been argued that the labeling requirements affect consumers’ risk perceptions of GM [2, 3].

Consumer acceptance and decision making related to food technology do not occur in a vacuum. The food chain involves a large number of interdependent actors who interact in order to provide food to consumers as end-users. Therefore, prior actions taken by food value chain (FVC) actors establish contextual factors for choices and decisions taken by the consumer. Grunert, Bredahl, and Scholderer [4] sketched an interdependency of decision making of FVC actors in the GM food context and concluded that there is no unanimous acceptance of, or expectations on, GM food among FVC actors. Importantly, this diversity of GM food stances by FVC actors may therefore induce different responses, with consequences for perceived risks among consumers. In fact, consumer decisions should be considered in a dependent system where the possible actions taken by FVC actors affect consumer decision making [5]. The question is how regulation of new food technologies affects the share of risk responsibility attributed to the self among consumers, and this is particularly relevant in the case of genetically modified (GM) food, given that consumers may be in an environment where they can control their choice of GM food. The connection between risk and responsibility then calls for further investigation of the cognitive process behind risk responsibility.

There is extant research about consumer acceptance of food biotechnology, in particular GM foods, using acceptance/rejection as the endpoint of decision making [see 6–8]. Furthermore, risk perceptions have been identified as an important determinant of consumer acceptance of the GM technology [6, 7, 9]. Extant literature suggest that health concerns influence the perceived risk of GM products [10], and that food safety and environmental concerns influence consumer acceptance of GM food [11, 12]. However, little is known about how consumers take responsibility for the risks that they identify, and whether this is affected by the regulation in place. Likewise, there is little evidence on consumers’ attribution of responsibility for these risks to themselves and to FVC actors, given the prevailing policy.

The issue of risk responsibility is central to consumer research and policy related to new food technologies because consumers do not choose the technology as such. Depending on the policy design, they may, however, be able to choose (or reject) food that involves the use of the technology. If there is a choice to be made, the consumer may attribute the risk responsibility to the self and/or to other upstream actors. The latter may be because consumers consider certain types of risk to be outside of their control, or because consumers consider it relevant to balance risks between themselves and other upstream actors. Following the literature on precaution by Gilovich and Medvec [13], this balance may differ depending on how the regulation of the food technology in question is designed, and to what extent risks are recognized by consumers. In this respect, there has been little research on the manner in which cognitive elements of relevance for information processing and deliberation relate to consumers’ own risk responsibility. An exception is a study by Leikas, Lindeman, Roininen, and Lähteenmäki [14], that examined the influence of food risk type (including the risk of consuming GM food) and risk perception on risk responsibility judgment.

Previous work has identified volition and control as two dimensions of self-control [15, 16]. Volition reflects the extent of belief in one’s ability to exert action to accept or avoid risk, and control over the outcome [15]. Volition thus relates to the nature of exposure to the risk, i.e., whether it is voluntary or involuntary exposure, and is therefore connected to Bandura’s [17] self-efficacy beliefs. Control, on the other hand, deals with the possibility of preventing an adverse outcome to oneself once the risky behavior has been initiated, independent of the perception of whether or not exposure to the risk is voluntary [15, 16]. In the context of risk, people obviously tend to prefer controllable over uncontrollable risks [18]. According to Nordgren *et al*. [15], appraisal of control diminishes perceptions of risk, whereas volition reinforces perceptions of risk.

The objective of this study is to examine whether the attribution of self-risk responsibility varied between four GM policy regimes, ranging from the strictest regulation to a full market access scenario. In particular, we examine whether this direct effect is mediated by perceived risk or self-control, respectively. We also explored if these effects were moderated by type of risk (environmental, health, socio-economic, or ethical risks). Through a field experiment, our study was designed to relate consumers’ attribution of risk responsibility to actions, specific to each scenario, taken by upstream FVC actors.

To the best of our knowledge, the relationship looked at here between risk responsibility and policy regime and actions by FVC actors in the context of GM food has not been addressed in previous research. The present study provides three contributions in relation to GM food risk responsibility. First, while the policy design has an effect on perceived risk and self-control, these effects do not transfer into self-risk responsibility. Our findings thereby draw attention to the connection between policy design and perceived risk. We found that consumers attributed less responsibility for GM food risks to themselves in comparison with upstream actors in the food value chain. However, consumers were still willing to take on non-negligible levels of responsibility for managing risks related to GM food. Second, and unexpectedly, we found a positive serial relationship between perceived risk and self-control across all policy scenario treatments. A potential explanation relates to the distinction between the control and volitional dimensions of risk. Third, we found that the main concerns among consumers were related to health risks, followed by environmental risks, while socio-economic and ethical risk were least commonly rated as the main concern. Our findings have implications for policy development regarding GM food and, in particular, risk management. In addition, the results obtained in this study shed light on the academic discussions around risk and responsibility associated with the development of biotechnology.

## 2. Background and research hypothesis

### 2.1. Theoretical background

Risk is a social construct phenomenon [19–24], and individuals’ perception of risk may be influenced by the situation and the regulatory context [refer to 25]. This implies that the regulation governing GM food production and market availability establishes a context that, in turn, may affect consumers’ perception of risk regarding GM food. This study examined how perceived risk and self-control serially mediate the causal relationship between the policy context and self-risk responsibility. Both perceived risk and self-risk responsibility are most commonly influenced by cognitive processing of information provided by other actors and deliberations related to one’s own situation [26]. The literature to date has addressed the relationship between perceived risk and self-control [27, 28], but there have been few systematic studies on how perceived risk relates to attribution of self-risk responsibility.

Findings in hazard research suggest that the extent of self-risk responsibility is lower in situations where the risk is perceived to be elevated. In These situations, others are expected to be more responsible instead [29, 30]. Mulilis and Duval [31] and Lalwani and Duval [32] provide substantial evidence that high perceived risk increases individuals’ level of self-risk responsibility (for their natural disaster preparedness), but only when the perceived resources are sufficient (relative to the magnitude of risk) to cope with the risk.

The literature has identified self-control and perceived risk as cognitive determinants of risk responsibility. Moreover, cognitive processing has been found to be risk type specific. Consumers’ risk perception of the introduction of GM food can be categorized into four major groups: human health and food safety, environmental, socioeconomic, and ethical risks. Despite their importance, cognitive elements of information processing and deliberation related to consumers’ own risk responsibility are not yet well understood.

The attribution, and distribution, of risk responsibility to the self and between the self and relevant others has been found to be affected by perceptions of personal controllability concerning food risks [33, 34]. There is evidence to suggest that manufactured risks are viewed as more controllable than natural hazards, and that controllability affects respondents judgment [14]. Therefore, perception of man-made risks, such as GM food, could affect responsibility judgments. Leikas *et al*. [14] found that personal responsibility judgment regarding different forms of food related risks, including GM food, was primarily predicted by people’s subjective evaluation of controllability.

Self-control refers to individuals’ judgment of the degree to which they perceive they can control the risk and its consequences when assessing risk [27, 28]. Controllability is considered to be an antecedent of personal responsibility [see 14, 34]. In forming behavior, self-control also relates to one’s belief in the ability to exert action and, furthermore, to the extent to which one can avoid an adverse outcome [35]. It is also closely related to the Heider [36] concept of controllability in the realm of attribution theory. Attribution theory deals with how people attribute causal explanations to events and controllability concerns whether a person perceives themselves to be actively in control of the cause [37].

Cognitive processing has been found to be risk dimension-specific [14]. Environmental risks of GM food are perceived differently from health-related risks [38]. Such differences may be manifested in the form of giving different weight to perceived risk components (likelihood and severity). Environmental risks of GM crops are characterized by low perceived control, whereas dietary risks (health) bring a greater perception of control [39, 40]. Leikas *et al*. [14] also showed that different food related risk types evoke different thoughts about one’s own responsibility.

As shown in Table 1, the literature on consumer attitudes and perceptions regarding GM food reports that consumer concerns fall into four major dimensions: human health and food safety risks, environmental risks, socio-economic risks, and ethical concerns [9, 41–44]. The human health risk dimension includes concerns regarding toxicity, allergenicity, nutrition effects, and long-running unknown effects [45, 46]. The environmental risk dimension refers to anxieties related to loss of biodiversity, gene escape and persistence, chemical use, and unexpected effects [44, 47, 48]. Socio-economic risks refer to farmer and industry rights, monopolies, freedom of choice, and public welfare [41]. The ethical risks reflect the conflicting religious values and perceptions of ‘unnaturalness’ associated with GM food [42, 43].

**Table 1:** Risk dimensions associated with adoption of biotechnology in food production.

Human health risks	Environmental risks	Socio-economic risks	Ethical risks
◾ Toxicity [49] ◾ Allergenicity [42] ◾ Unknown risks [45, 50]	◾ Biodiversity loss [48, 51] ◾ Gene drift [42] ◾ Gene persistence [52] ◾ Non-target species effects [47] ◾ Increased use of chemicals [48, 51] ◾ Uncertainties regarding long-term effects [53–55]	◾ Intellectual property rights [42] ◾ Farmers’ rights [51, 56] ◾ Monopolies [57] ◾ Reduced freedom of choice [55] ◾ Public welfare debate [41]	◾ Conflicting religious values [42, 58] ◾ Tampering with ‘God’s plan’ [43] Unnaturalness [43, 55, 59, 60]

### 2.2. Research hypotheses

**Fig 1:**
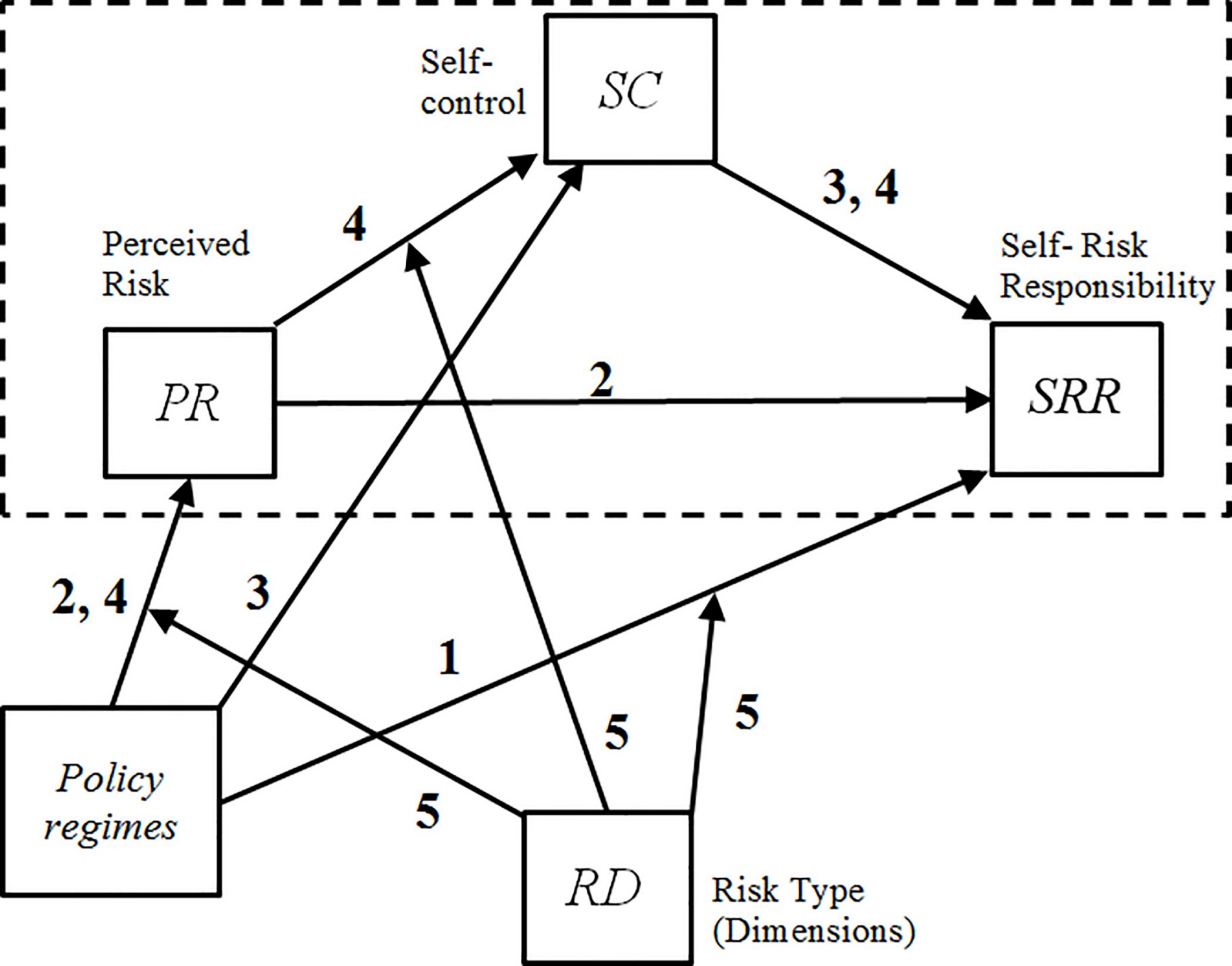
Conceptually moderated mediation model for estimating the effect of Policy Scenario on self-risk responsibility (SRR) judgment through perceived risk (PR) and self-control (SC) depending on risk dimension (RD). Note: the dashed line depicts the elements of the conceptual model that refer to cognitive processing of information and deliberations on one’s own situation.

Based on the above discussions, Fig 1 outlines the conceptual model used for determining the effect of policy scenarios on self-risk responsibility through perceived risk and self-control. The risk dimensions (RD) are modelled as a moderator, meaning that the effect of the policy scenario on self-risk responsibility may depend on the characteristics of the moderator. This analysis can therefore establish the boundary conditions, or the circumstances, in which changes in policy relate to risk responsibility.

Based on the conceptual model in Fig 1, five main hypotheses were tested in this study:

**H**_**1**_: The policy scenario is a direct determinant of consumers’ attribution of self-risk responsibility (*SRR*) (path (1) in Fig 1).
**H**_**2**_: Perceived risk mediates the relationship between the policy scenario and *SRR*. (path (2)).
**H**_**3**_: Self-control mediates the relationship between the policy scenario and S*RR*. Lower self-control (*SC)* decreases *SRR* (path (3)).
**H**_**4**_: The effect of the policy scenario on *SRR* is mediated through perceived risk (*PR*) and through SC (path (4)).
**H**_**5**_: There is a total effect of policy on self-risk responsibility, including both a direct (H_1_) and indirect effect (H_2_-H_4_) through *PR* and the judgment of *SC*.

In the absence of theoretical grounds to the contrary, all cognitive components for the model (*PR*, *SC*, and *SRR*) were hypothesized to be moderated by risk dimension (*RD*) (path (5)).

## 3. Material and methods

To test the above listed hypothesis, a field experiment (FE) was conducted. Plott [61] pointed out that “*while laboratory processes are simple in comparison to naturally occurring processes*, *they are real processes in the sense that real people participate for real and substantial rewards and follow real rules in doing so*. *It is precisely because they are real that they are interesting*.” An important advantage with FEs over conventional laboratory experiments is that it draws on non-student participants [62, 63]. Thus, the use of a FE in the present case allowed control over the laboratory environment and involved participants identified as being responsible for their household’s food purchases as consumers [64].

### 3.1. Recruitment and participants

Participants were recruited through a postal invitation sent out to a sample of 7,000 randomly selected residents (by Statistics Sweden) aged 18–75 years in Uppsala municipality. Formal ethical approval was exempted in accordance with the Swedish Ethical Review Act (2003:460) regarding our research, because it does not subject respondents to potential harm or risk. The invitation card briefly indicated that the study related to food choice and food production, but the focus of the study (i.e. GM food) was not mentioned, to avoid self-selection bias [65]. A reminder was sent out after one week. People who were interested in participating signed up using an online form posted on the university website and obtained a randomly generated unique code to participate anonymously (after signing an online consent form). The registration was comprised of a questionnaire to collect data on participants’ socio-economic characteristics. Participants were able to select a session time to take part in the experiment (see Appendix I, Box 4 in S1 File). The experiments were carried out at the computer labs in the Uppsala University. Participants were able to leave the experiment at any stage. Participants could also ask questions during the sessions. As compensation, they received a gift card (for the grocery store of their choice, or a movie theatre ticket) to the value of SEK 300, upon completion of the study.

The total number of participants was 547. Data from 12 participants was discarded due to incomplete answers, or as requested by the participants. Although participants had a broad range of age and education level and were of mixed gender, the sample was not representative of the Swedish population with respect to these criteria. However, the differences between the sample characteristics and the Swedish population at large are reasonable, given that Uppsala is a major university city. A summary of demographic sample characteristics is presented in Appendix IV (Table D1) in S2 File. Tests for treatment-specific selection bias were not conducted, since the net difference in observable causes (gender, education, income, age, and food purchasing responsibilities) was expected to be zero at the baseline, given the randomization of the scenarios [66].

### 3.2. Treatments and experimental procedures

In June and August 2014, a total of 30 sessions were held and one of the authors attended all the sessions as the experimenter. In order to have better control over the experiment process, only 20 participants were allowed to participate in each session. The respondents were randomly assigned to one of four treatments, representing different policy scenarios (i.e., *Banned*, *R&D*, *Import*, *Full)* (Appendix III in S2 File). In all other respects, the participants were treated equivalently, and all procedures were identical in all experimental conditions.

The *Banned* scenario referred to a policy setting in which all activities related to production and commercialization of GM food were prohibited. The *R&D* scenario depicted a policy where research activities on applications of GM to food production were allowed, but not importation, cultivation, or commercialization of GM foods. In the *Import* scenario, imported GM food was allowed on the market alongside non-GM food with a mandatory labelling policy, but there was no domestic production. Finally, in the *Full* scenario, GM food was available on the market, with a mandatory labelling policy, and domestic production was allowed.

The participants were instructed to take the role of a consumer during the session in which they participated. Each session lasted about 70 minutes and was started by welcoming the participants, providing information about data confidentiality and procedures, and information about the structure and main roles of food value chain actors, so as to set a common frame for the experiment (see Appendix IV in S2 File). Information about biotechnology and its potential applications, as well as the relevant supply chain was also provided (Appendix II, S3 Fig in S1 File).

Following a brief description of the policy scenario, participants were asked to rank the four risk dimensions (health, environment, socio-economic, and ethical) according to their relative importance (see Appendix II, Box 2 in S1 File): *Which of the following aspects are most relevant to you for the evaluation of the above [policy] scenario*? *Rank 1–4* (where 1 = least relevant and 4 = most relevant).

Based on the ranking results, a set of risk statements pertinent to the risk dimension and policy context (treatment) were presented to respondents. The extensive literature review by Hess et al. [67] was used to identify statements that captured risks relevant to each risk type and with reference to the regulatory context associated with policy scenarios. Risks attributable to individuals, certain groups, or society were developed into risk statements that expressed either direct or indirect risks. For each policy scenario, the statements covered a broad range of documented risks, such that the statements could be understood as formative indicators from a scale-development perspective. The final set of statements included 3–4 statements for each combination of risk dimension and policy scenario (see S7 File). Respondents were then asked to indicate whether they agreed with the risk statement, the likelihood of its occurrence, degree of its severity and controllability (see section 2.3).

### 3.3. Eliciting risk responsibility and perceive risks

Rayner and Cantor’s [68] approach was adopted for measuring perceived risk. However, the variable *agreement* was added in the risk measure to enhance construct validity. Accordingly, participants were first asked if they agreed with the relevance of the specific risk statement on a 5-point scale (0 = *totally disagree*, 0.25 = *disagree*, 0.5 = *neither agree nor disagree*, 0.75 = *agree*, and 1 = *totally agree*). They were next asked to indicate the perceived likelihood of the respective statements on a 6-point scale (1 = *not likely at all* to 6 = *almost entirely certain that this will happen*). They were then asked to rate the severity of the event, if it was to occur, on a 5-point scale (1 = *not severe at all* to 5 = *very severe*). Participants were presented with a table that provided an overview of their responses related to agreeableness, likelihood, and severity, and then asked to judge how confident they were about their assessment. Because of the correlation between the three components, the overall index for perceived risk *(PR)* for each participant was computed as the product of agreement (*A*_*i*_|∀ *A*_*i*_≠0,*i* = 1,2,3,…*I*), likelihood (*L*_*i*_), and severity (*S*_*i*_) scores, such that:

$${PR}_{jkt}=\prod_{i=1}^{I}{\left[A_{i}(L_{i}\times S_{i})\right]}^{\frac{1}{I}}$$ (1)

where *j* denotes participant (*j* = 1, 2, 3, … *J*), *i* is the valid (i.e., non-zero agreement) statement (*i* = 1, 2, 3, … *I*) relevant to each risk dimension (*k* = 1, 2, 3, 4), and *t* is the policy scenario (*t* = 1, 2, 3, 4). The *PR* index was normalized to the power of one over the total number of valid statements (*I*) in each risk dimension. The agreement multiplier served as a validity check of the statements in a way that excluded *L*_*i*_ and *S*_*i*_ responses on statements with which the participant totally disagreed (*A*_*i*_ = 0), but multiplied *A*_*i*_, *L*_*i*_, and *S*_*i*_ for statements that had non-zero agreement. The maximum level of perceived risk is: *PR* = 1×(6×5) = 30. Likewise, the normalized Self-Controllability index (*SC*_*jkt*_) was determined with the multiplication of scores on controllability (*C*_*i*_) by the agreement with each statement (*A*_*i*_):

$${SC}_{jkt}=\prod_{i=1}^{I}{\left[A_{i}\times C_{i}\right]}^{\frac{1}{I}}$$ (2)

where *C*_*i*_ is the score on controllability measure given to each risk statement on a 5-point scale (1 = *I have no control at all over my exposure to this risk*, 5 = *I can completely control my exposure to this risk*).

The risk responsibility measure was elicited by asking participants to indicate the share of responsibility attributed to themselves and other actors in the food value chain (Appendix II in S1 File). Following Leikas *et al*. [14], we asked “*Please use the table below to allocate your view on the share of responsibility that each decision-maker in the food chain should have*. *Think of yourself when filling in the extent to which consumers are responsible*”. Using a constant-sum scaling of feature importance [e.g. 69], participants were given 100 points to distribute across food value chain actors, to indicate the share of responsibility given to each actor type. Participants were allowed to adjust and verify the distribution of their points before completing the task. The analysis in this study considers self-risk responsibility (SRR in Fig 1), and thereby includes only the share that participants attributed to themselves as consumers.

## 4. Data analysis

### 4.1. Descriptive statistics for risk responsibility, risk dimensions and perceived risk

Descriptive statistics and all data visualizations were prepared using an R statistical software package version 3.12.1 [70], ‘Dunn test’ package version 1.3.5 [71], and ‘ggplot2’ plotting package version 1.0.1 [72].

**Fig 2:**
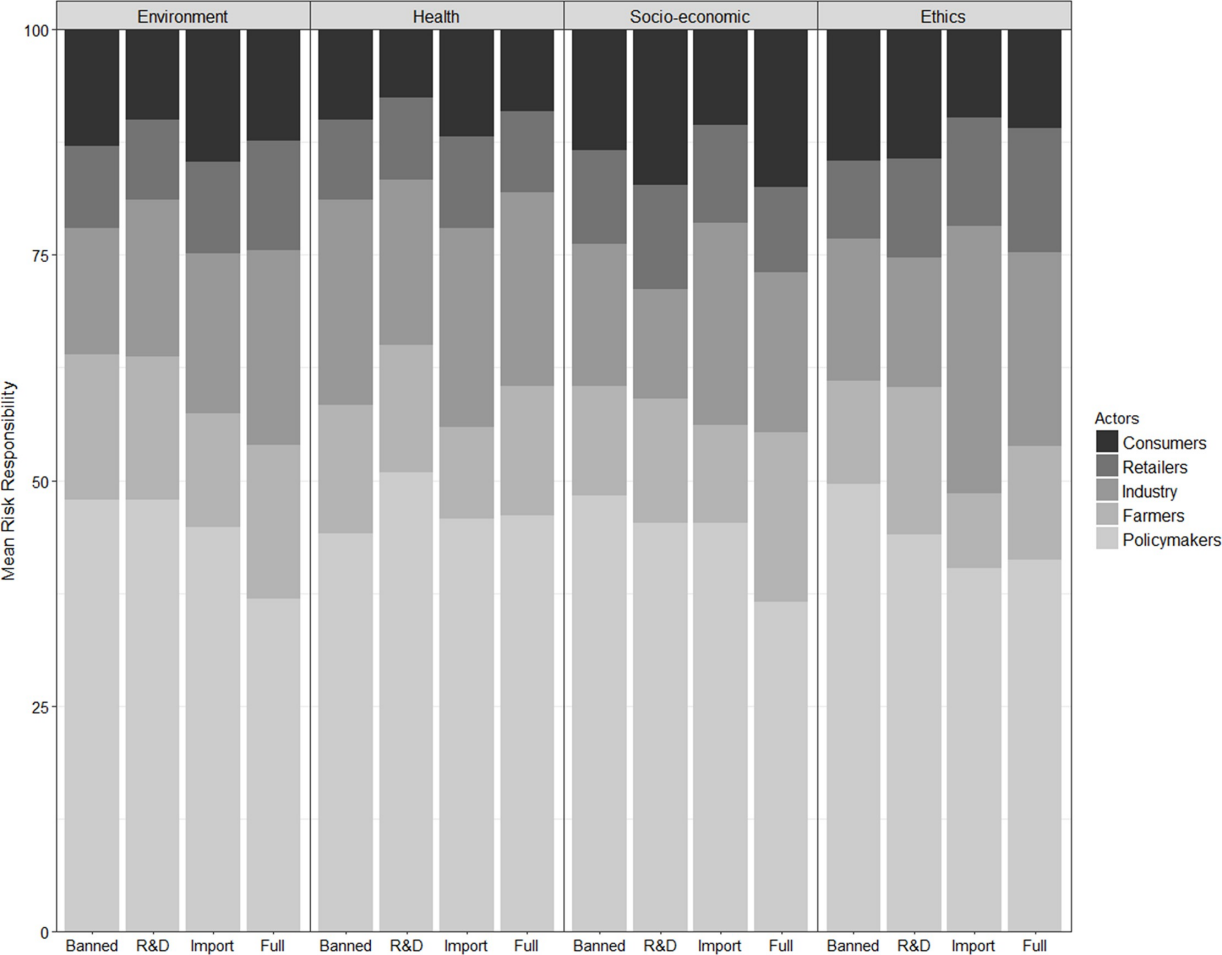
Share of mean risk responsibility over policy scenarios attributed to food value chain actors.

As Fig 2 shows, the highest share of responsibility was assigned to public authorities, followed by the food processing industry (overall average *RR*_policymakers_ = 45.39%, *RR*_industry_ = 19.6%). These findings are consistent with those of Leikas *et al*. [14] and Van Wezemael, Verbeke, Kügler, de Barcellos, and Grunert [73]. The average share of risk responsibility attributed to the self was 11.4% among participants (Appendix VI, Table F3 in S3 File). We also note that the attribution of risk responsibility to different food value chain actors differed between policy scenarios ($x_{Freidman-test}^{2}=909$, df_k-1_ = 4, P<0.001); most notable are public authorities followed by industry (Appendix VI, Box F2 in S3 File). However, the average share of risk responsibility attributed to retailers was no different from those attributed to the self (Wilcoxon pairwise test: p-value = 0.08 adjusted using Holm [74]).

In the remainder of the analysis, we focused on the risk responsibility that the participants attributed to the self (*SRR*). Kruskal-Wallis rank sum test indicated that there is no significant difference in the SRR across scenarios, however SRR is attributed significantly lower (mean = 9.6, median = 10) for those with concerns of health risks (nonparametric Kruskal-Wallis test chi-squared = 21.4 df = 3, p-value<0 adjusted using Holm [74] and post hoc Dunn test with Bonferroni adjustment) (See Appendix VI, Table F6 in S3 File).

Table 2 shows the perceived risk score across policy scenarios and risk types. A total of seven participants were discarded from the dataset at this point because they totally disagreed with all risk statements, which left a final sample size of 528. Across all scenarios, health risks were the most relevant risk type (n = 252), followed by environmental risks (n = 141), ethical risks (n = 74), and socio-economic risks (n = 61).

**Table 2:** Mean perceived risk and number of participants (n) in each policy scenario-risk type dimension.

	Policy scenario				
Risk Dimension	Banned	R&D	Import	Full	Total
Environmental	7.10	11.30	7.31	16.24	10.4
	(35)	(34)	(36)	(36)	(141)
Health	6.18	7.54	11.73	11.81	9.4
	(52)	(68)	(66)	(66)	(252)
Socio-economic	8.90	6.67	8.63	10.48	8.7
	(12)	(16)	(17)	(16)	(61)
Ethical	4.66	8.59	11.95	12.00	8.8
	(25)	(15)	(17)	(17)	(74)
Total	6.1	8.4	10.2	12.9	9.5
	(124)	(133)	(136)	(135)	(528)

Note: The maximum PR is 30 for each individual as it is normalised based on number of statements (see Eq 1).

Also, results indicated that the highest magnitude of perceived risk was related to environmental (PR_Env._ = 10.4) and health concerns (PR_Health_ = 9.4). The results of Kruskal-Wallis tests ($x_{Env.}^{2}=10.93$, df = 3, P = 0.0121; $x_{Health}^{2}=61.08$, df = 3, P<0.001; $x_{Ethical}^{2}=27.93$, df = 3, P<0.001) indicated statistically significant differences in the risk perception under different policy treatments and due to different risk concerns, except for socio-economical risk ($x_{socio.}^{2}=3.46$, df = 3, P = 0.3) dimension (see Appendix VII, Table H4 in S5 File).

Among the participants who rated environmental risk with GM food as their main concern, the perceived risk was highest in the *Full* and *R&D* policy scenarios. Both scenarios allowed for domestic cultivation of GM food, although the *R&D* scenario only permitted GM crops to be grown on a limited scale in research field trials. For participants who held health risks as their main concern, the perceived risks were highest in the *Import* and *Full* policy scenarios, in which GM food was available for consumption. Interestingly, for participants who indicated socio-economic risks as their main concern, *PR* was higher in the *Banned* scenario than in the *R&D* scenario. This suggests that specific risks related to socio-economic opportunity losses were more relevant in a situation where GM technology was inaccessible. Furthermore, perceived risk was highest in the policy scenarios where GM food was available (*Import* and *Full*) among participants indicating ethical concerns as most important.

Moreover, the Kruskal-Wallis analysis indicated significant differences in self-control perception of different risks (Kruskal-Wallis chi-squared = 27.87, df = 3, p-value < 0.001). Then, post hoc analysis (Bonferroni P-value adjusted = 0.025) confirmed the significant differences between mean SC environmental-health and environmental-socio-economic risk dimensions (Appendix VIII in S4 File). However, different policy treatments did not induce different self-control perception, as determined by one-way ANOVA (F (3,524) = 0.03, p = .99).

### 4.2. Results from the moderated mediation analysis

We analyzed whether the attribution of self-risk responsibility (SRR) varied between policy scenarios (PS), and if such an effect was mediated by perceived risk (PR) or self-control (SC). We also explored whether these effects depended on the type of risks (RD). Mediation analyses were conducted using IBM SPSS statistical software package version 22 [75] and PROCESS macros developed by Hayes [76, 77]. The PROCESS syntax was adapted by allowing for multiple mediators (details available from the authors upon request).

As the policy scenario variable was multi-categorical (*Banned*, *R&D*, *Import*, and *Full*), a dummy-coding for T group of policy scenarios (*PS*_*t*_, *t* = 1,…, *T*-1) was deemed appropriate, with *PS*_*t*_ set to 1 if a participant was in scenario *t*, and 0 otherwise [77]. The moderator (risk dimension) was multi-categorical, and *PR* and *SC* related to these risk dimensions derived from non-identical statements (see Appendix in S1 File). Hence, the dataset was divided into four sub-datasets (Model 1 = environmental risks, Model 2 = health risks, Model 3 = socio-economic risks, and Model 4 = ethical risks) in order to evaluate the moderating effect of risk dimension in the sub-models.

**Fig 3:**
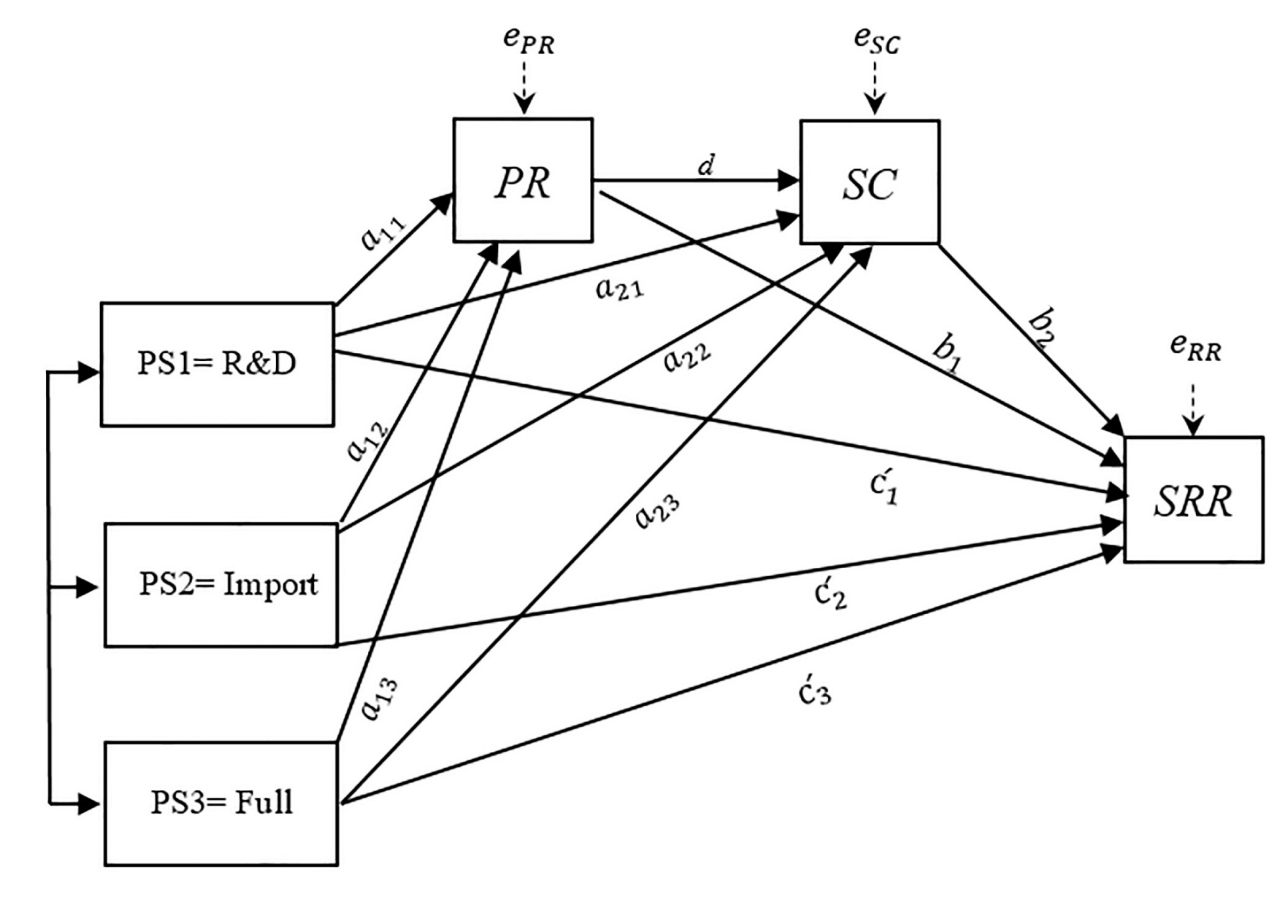
Statistical model for estimating the effect of Policy Scenarios (PS_t_) on self-risk responsibility (SRR) through perceived risk and self-control. The model is adapted from Hayes and Preacher [77] and Hayes [78]. Socio-demographic factors and moderator risk dimension (RD) are excluded from the figure to reduce visual clutter.

The statistical diagram in Fig 3 represents the structure in which the policy scenario operates directly and indirectly on *SRR* through *PR* and *SC*. The model includes all possible indirect effects between policy scenario treatments and self-risk responsibility and allows for the effects of treatments on mediators (perceived risk and self-control), and of mediators on *SRR*. Following Hayes and Preacher [77], since the policy treatment was multi-categorical, the effects should be interpreted relative to the reference group. The relative direct effect of policy scenarios quantified how much the mean difference between policy scenarios (*PS*_*t*_) were expected to differ on self-risk responsibility, independent of the mediator effects. The relative indirect effect was interpreted as the mean difference between scenarios that were estimated to differ on *SRR* as a result of the effect of *PS*_*t*_ on mediators (i.e., *PR* and *SC*) which in turn affects *SRR*. The total relative effect of *PS*_*t*_ on *SRR* was taken as the effect of mean differences between scenarios estimated to differ on *SRR* through both the direct and indirect pathways.

The model in path diagram form depicted in Fig 3 translates to three linear equations:

$$PR=\delta_{PR}+a_{11}{PS}_{1}+a_{12}{PS}_{2}+a_{13}{PS}_{3}+\sum_{k=1}^{K}\gamma_{k}U+e_{PR}$$ (3)

$$SC=\delta_{SC}+a_{21}{PS}_{1}+a_{22}{PS}_{2}+a_{23}{PS}_{3}+\sum_{k=1}^{K}\gamma_{k}U+dPR+e_{SC}$$ (4)

$$SRR=\delta_{SRR}+\overset{´}{c_{1}}{PS}_{1}+\overset{´}{c_{2}}PS+\overset{´}{c_{3}}{PS}_{3}+b_{1}PR+b_{2}SC+\sum_{k=1}^{K}\theta_{k}U+e_{SRR}$$ (5)

where *δ*_*PR*_, *δ*_*SC*_ and *δ*_*SRR*_ are regression intercepts and *e*_*PR*_, *e*_*SC*_, and *e*_*SRR*_ are errors in the estimation of *PR*, *SC*, and *SRR*. A general linear modelling approach was adopted to estimate the direct and indirect effects on Eqs 3–5 [76, 77, 79]. The analysis was conducted using log transformation of data, as the mediators and the outcome variable were not normally distributed.

The main variables of interest are the $\overset{´}{c_{t}}$− coefficients in Eq (5), capturing the differences between policy scenarios in *SRR* holding mediators constant. The two *b*-coefficients refer to the effects of mediators PC and SC on S*RR*, while statistically equating the policy scenario treatments *PS*_*t*_ on average. The parameter *d* (Eq 4) captures the effect of *PR* on *SC*. For each *PS*_*t*_ there were three relative indirect effects on S*RR* through *PR* and *SC* in each model (i.e., nine total indirect effects). According to Hayes [78], the product of the paths (and not the paths themselves) defines the indirect effects. In each *PS*_*t*_ the total effect (*c*_*t*_) on *SRR* was obtained as the sum of all relative direct and indirect effects, ($c_{t}=c_{t}^{\prime }+a*b$). The total effect quantified mean differences in *SRR* between scenarios. *U* is a set of socio-demographic dummies describing the gender, education level, age and income level of the participants, and γ and θ are parameters to be estimated. Details on these variable specifications are provided in Appendix V in S2 File.

To test for relative indirect effects, following Hayes and Preacher [77] a *percentile bootstrap confidence interval* (*CI*) was constructed by repeatedly taking samples with replacement, and estimating all the coefficients in the mediation model using Eqs (3), (4) and (5) in each bootstrapped sample. Using an approach with 25,000 iterations, the distribution of relative indirect effects served as empirical approximations of sampling distributions. As a series of analyses were conducted on the same data, the significance level was adjusted using a Bonferroni correction [77]. The adjusted significance level was α = 0.025. The 97.5% *CI* for each relative indirect effect was computed as the bootstrap estimates defining the lower and upper 97.5% of the distribution. The relative indirect effect is deemed statistically significant if the *CI* does not straddle zero [77].

**Fig 4:**
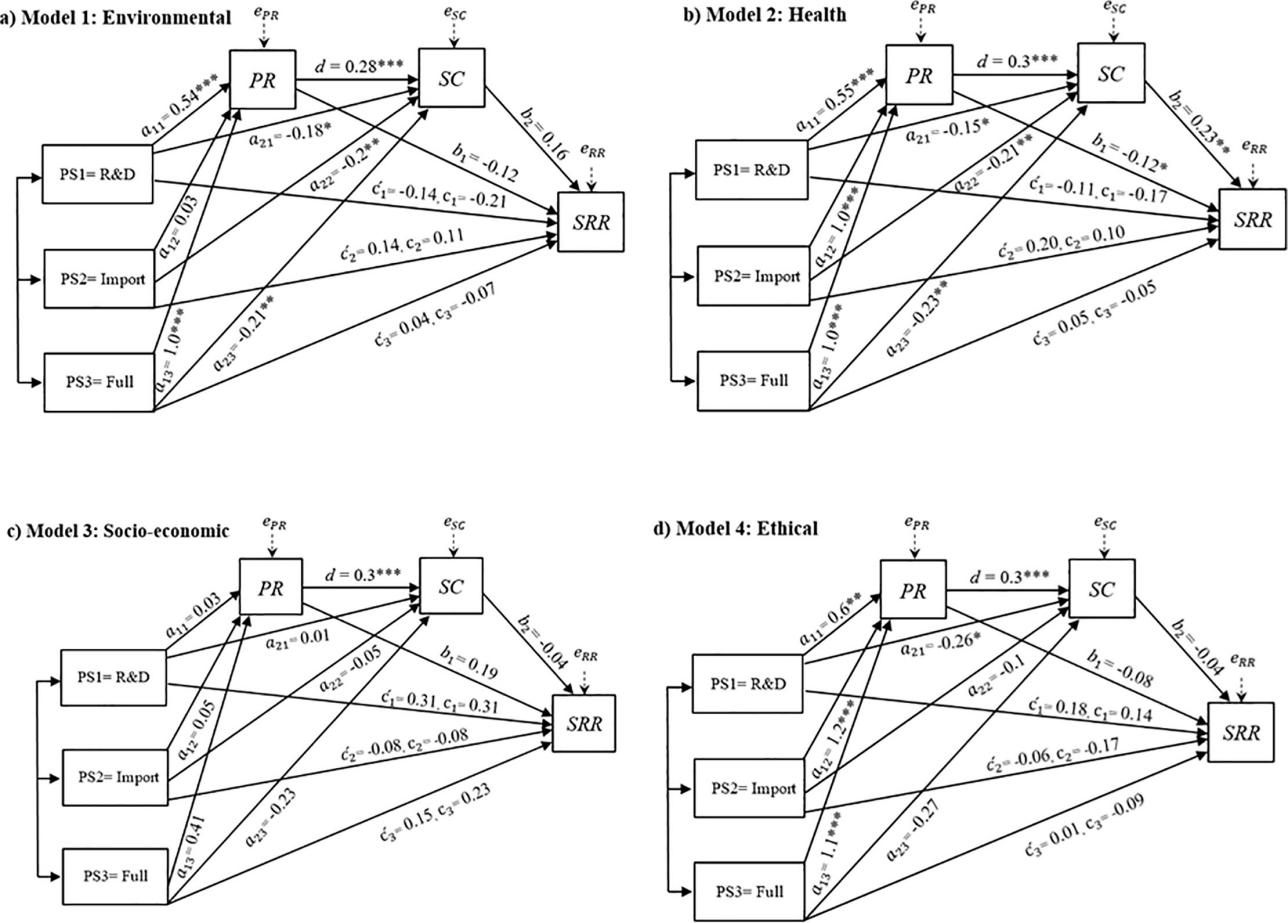
Estimated model coefficients. Note: *P < .1, **P < .05, ***P < .01. Estimates for the socio-demographic variables are excluded from the figure to reduce visual clutter. Estimates for all coefficients related to model two are available in Appendix V (Table E1) in S2 File.

Fig 4 shows the relative direct, indirect, and total effects from the moderated mediation analysis. As shown in Fig 4A–4D, none of the relative direct effects ($\overset{´}{c_{t}}$) were significant. Therefore, hypothesis 1 was not supported; there was no significant effect of GM policy (*PS*_*t*_) on *SRR* after accounting for *PR*, *SC*, age, gender, income, and education.

Next, we tested if the impact of policy scenario on self-risk responsibility was mediated by perceived risk or self-control (H_2_-H_4_). First, we note that in all models in Fig 4, *a*_11_, *a*_12_, and *a*_13_ correspond to the mean differences in perceived risk between the *R&D*, *Import*, and *Full* policy scenarios, respectively, relative to the reference scenario (*Banned*). All policy scenario coefficients were significant in Models 1, 2, and 4 (except *Import* in Model 1). The sign of the coefficients indicated that relaxing the regime by allowing research-related cultivation (*R&D*) was associated with a higher perceived risk compared to the *Banned* scenario. Models 2 and 4 showed that allowing GM products on the market (*Import* and *Full*) was associated with even higher levels of perceived risk, while there was no significant difference in the perceived risk depending on whether domestic production was allowed (*Full*) or not (*Import*).

In Fig 4, *a*_21_, *a*_22_, and *a*_23_ correspond to the mean differences in self-control perception between the *R&D*, *Import*, and *Full* policy scenarios, respectively (relative to the reference *Banned* scenario). These coefficients were negative and statistically significant in Models 1 and 2, implying that the relaxed policies (*Import* and *Full* scenarios compared to *Banned*) decreased Self Control.

The *b*_*1*_ and *b*_*2*_ –coefficients represent the mean differences in *SRR* with respect to the changes in mediators. These coefficients were only statistically significant in Model 2 (health risk), where an increase in *PR* implied a decrease in *SRR*, while an increase in *SC* implied an increase in *SRR*. In all models, *PR* was positively linked to *SC* and was statistically significant (coefficient *d*).

The indirect effects of interest for testing H_2_-H_4_ were estimated for each model separately. Bootstrapped *CI* hypothesis tests showed that only in Model 2 (health) were the relative indirect effects statistically significant, and we therefore proceed with results for this model only. As expected, Model 2 showed the most statistically significant parameters, noting that the number of observations in this model was higher (252 compared to 141, 61 and 74 in Models 1, 3 and 4, respectively). As presented in Table 3, bootstrapped confidence interval estimates for *PR* on *SRR* straddled zero for all policy scenarios. Therefore, hypothesis 2 could not be supported. This does not support earlier findings in the field of natural hazard research, in which an inverse relationship between perceived risk and self-risk responsibility has been reported [see 29, 30].

**Table 3:** Relative indirect effects related to health risks dimension (Model 2).

Indirect effects key	Relative indirect effect estimates	Boot LL*CI*^1^	Boot UL*CI*^2^	Test of Hypothesis
Ind_1_*: R&D →PR→RR	*a*_11_×*b*_1_ = 0.5499 × (-0.1234) = -0.07	-0.194	0.025	H_2_
Ind_2_: Import →PR→RR	*a*_12_×*b*_1_ = 1.0129 × (-0.1234) = -0.13	-0.319	0.056	H_2_
Ind_3_: Full →PR→RR	*a*_13_×*b*_1_ = 0.9717 × (-0.1234) = -0.12	-0.319	0.053	H_2_
Ind_4_: R&D →SC→RR	*a*_21_×*b*_2_ = (-0.1526) × 0.2316 = -0.04	-0.118	0.005	H_3_
Ind_5_: Import→SC→RR	*a*_22_×*b*_2_ = (-0.2121) × 0.2316 = -0.05	-0.141	-0.002	H_3_
Ind_6_: Full→SC→RR	*a*_23_×*b*_2_ = (-0.2266) × 0.2316 = -0.05	-0.147	-0.003	H_3_
Ind_7_: R&D →PR→SC→RR	*a*_11_×*d*×*b*_2_ = 0.5499 × 0.2975 × 0.2316 = 0.04	0.004	0.098	H_4_
Ind_8_: Import →PR→SC→RR	*a*_12_×*d*×*b*_2_ = 1.0129 × 0.2975 × 0.2316 = 0.07	0.007	0.159	H_4_
Ind_9_: Full →PR→SC→RR	*a*_13_×*d*×*b*_2_ = 0.9717 × 0.2975 × 0.2316 = 0.07	0.007	0.157	H_4_

^1^Bootstrapped lower-level confidence interval (97.5%)

^2^Bootstrapped upper-level confidence interval (97.5%)

*‘Ind’ refers to ‘indirect path’

The *CI* interval for indirect paths associated with the *Import* and *Full* scenarios to *SRR* via self-control excluded zero. These results confirm hypothesis 3 and verify earlier findings on the predictive role of *SC* for GM food risk responsibility [14, 80].

The relative indirect effects via paths 7, 8, and 9 had a positive sign, which suggests that the less restrictive GM policies increased the *PR*, which in turn increased *SC* and, consequently, *SRR*. These results support hypothesis 4, less restrictive policies (*Import* and *Full*) implied higher attribution of self-risk responsibility as a result of the serial mediating effects of *PR* through *SC*.

Finally, we estimated the total effects (*c*_*t*_) from policy scenarios on self-risk responsibility. As shown in Fig 4, these estimates were insignificant in all risk dimensions, lending no support for H_5_ regarding the existence of total effects on *SRR*.

Overall, the socio-demographic covariates (age, gender, education, and income) performed poorly as explanatory variables for differences in risk perception and self-risk responsibility attribution (Appendix V, Table E1 in S2 File). An exception was that higher age was associated with lower self-control and self-risk responsibility attribution. These results support previous findings of greater acceptance of GM food among young people [81, 82]. In addition, the coefficient for higher education was statistically significant, revealing a relatively positive effect of higher income level on self-risk responsibility, irrespective of mediator effects.

## 5. Discussion and conclusions

### 5.1. Key findings

The main question addressed in this study was whether the risk responsibility that individuals attribute to themselves is affected by GM policy design. We further examined whether this direct effect is serially mediated by perceived risk or self-control. We also tested if these effects were moderated by type of risk (environmental; health; socio-economic; or ethical risks).

We conducted an experiment, including four different GM policy scenario treatments. We found that consumers attributed less responsibility for GM food risks to themselves in comparison with upstream actors in the food value chain, in line with previous studies [e.g. 73, 83, 84]. However, consumers are still willing to take on non-negligible levels of responsibility for managing risks related to GM food. These findings are relevant for the design and effectiveness of risk communication in general, and labeling schemes in particular, in situations when GM food products are available on the market.

We found that the main concerns among consumers were related to health risks, followed by environmental risks, while socio-economic and ethical risk were least likely to be rated as the main concern. This supports the idea that health concerns influence the perceived risk of GM products [10], and that food safety and environmental concerns influence consumer acceptance of GM food [11, 12].

The hypothesized direct effect from GM food policy on self-risk responsibility was not supported in the data. However, we found that policy design influences perceived risk and self-control. In general, policy scenarios with GM food market availability implied that participants attributed higher levels of perceived risk and lower levels of self-control to GM food. A scenario where domestic research-related cultivation was allowed was associated with higher levels of perceived risk compared to scenarios where all domestic production was banned. The perceived risk was even higher when GM food was allowed on the market, independent of whether the products are imported or cultivated domestically.

Unexpectedly, we found a positive serial relationship between perceived risk and self-control across all policy scenario treatments. A potential explanation relates to the distinction between the control and volitional dimensions of risk. Previous work has identified volition and control as two dimensions of self-control [15, 16]. Volition reflects the extent of belief in one’s ability to exert action to accept or avoid risk, and control over the outcome [16]. Volition thus relates to the nature of exposure to the risk, i.e., whether it is voluntary or involuntary exposure. Control deals with the ability to prevent an adverse outcome to self, independent of whether the exposure is perceived as voluntary [15, 16]. In the context of risk, people obviously tend to prefer controllable over uncontrollable risks [18]. Moreover, acknowledgment of control over certain risks reduces perceptions of risk, while volition reinforces perceptions of risk [15]. A distinction between volition and control is recommended in future research on the effects of perceived risk and self-control as determinants of self-risk responsibility.

While we did not find evidence supporting a direct effect of GM food policy on self-risk responsibility, there was an indirect effect from policy scenarios on self-risk responsibility, as mediated by perceived risk and self-control among respondents who rated health risks as their main concern. Among these individuals there were two different cognitive mechanisms operating on self-risk responsibility. In one mechanism, less restrictive policies implied lower levels of self-control, which in turn reduced self-risk responsibility. In the other mechanism, both perceived risk and self-control mediated the effect on self-risk responsibility from the policy regime. These results show that GM food commercialization increased perceived health risks, which in turn implied higher levels of self-control and self-risk responsibility.

### 5.2. Main contributions and implications for various stakeholders

This study contributes insights into the effects of GM policy and type of risk on perceived risk, self-control and, ultimately, attribution of self-risk responsibility. Several of our findings are of direct relevance for risk communication and policy design. Firstly, although consumers attributed less responsibility for GM food risks to themselves in comparison with upstream actors in the food value chain, they are in fact willing to take on non-negligible levels of responsibility for managing risks related to GM food. This is relevant for the design and effectiveness of risk communication in general, and labelling schemes in particular, in situations when GM food products are available on the market.

Secondly, while the share of self-risk responsibility is not directly affected by policy design, our results show that policy design has an effect on perceived risk and self-control. Interestingly, the more stringent GM policies decreased the perceived risks and increase the perceived self-control. These findings contradict the theoretical arguments that stricter policies signal higher risks to consumers [2, 3, 85]. We note that these arguments regard the signal from labeling type (mandatory vs. voluntary), while the policy scenarios in this study are on the production and market availability side. Following the theoretical contributions on the effects of labeling policies, a recent study by Kolodinsky and Lusk [80] investigates how changes in GM labeling requirements in the state of Vermont, USA, affected attitudes. They found that, in line with the results of this study, the presence of GM labels serves to dispel consumer opposition towards GM food. Our findings thereby contribute to the discussion on the signals different policy regimes may be sending.

### 5.3. Limitations of the study and suggestions for future research directions

Although the overall findings suggest that policy design does not affect self-risk responsibility attribution, the connection cannot be ruled out as non-existent. Several features in this study may contribute to the insignificant results. Firstly, there are likely to be important differences for individuals between being presented a policy scenario in an experiment, on the one hand, or experiencing it in society on the other. The design of a policy is likely to be interpreted and discussed in media and social media, and among peers. For this reason, we might expect that the effects of policies are more pronounced in real settings compared to this study.

We also note that the elicitation of perceived risk and self-risk responsibility is based on questions concerning GM food in general. While this contributes to a generalizability of the results, it is also a limitation of the study. Indeed, as suggested by Frewer *et al*. [9] and Siegrist and Hartmann [86], we suspect that cognitive information processing may be differentiated in relation to different GM applications and product end-uses, because the underlying ethical or personal value system may act to confirm or oppose the use of biotechnology more for some uses than for others. Moreover, while we found indirect effects from policy scenarios on self-risk responsibility only among participants that considered the health risk dimension to be most important, it should be noted that the distinction between risk dimensions is not straightforward and, to some extent, they could be interrelated. For instance, Dowd and Burke [87] pointed out that ethical values affect individuals’ perception of environmental risk and drive their level of concern, especially among ecologically conscious consumers. Moreover, although we have found significant differences in risk perception across respondents with health, environmental, ethical, socio-economic concerns, lower samples of ethical and socio-ethical groups would undermine the validity of results [88].

Food policy development in Europe is seeing an institutional shift, in which the general public has a greater voice and its views are considered in the policy-making process [89]. For food policy development to be effective, consumer decision-making and risk responsibility needs to be well understood and addressed accordingly. The findings in this study reveal that, while the policy design has an effect on perceived risk and self-control, these effects do not transfer to self-risk responsibility (this interpretation should be done with care due to the non-representative sample obtained in this study). Our findings, therefor, draw attention to the connection between policy design and perceived risk, which in turn has been found to form the basis of the heuristic framework used to understand acceptance of GM food [90]. It has been argued for increased public engagement to be one of the constituent elements of the political discourse affecting policy development and implementation of emerging technologies [89, 91, 92]. The growing importance of public engagement in EU food policy development and the intimate connection between risk and responsibility call for further investigation of the cognitive process behind risk responsibility.

## Supporting information

S1 File (PDF)

S2 File (DOCX)

S3 File (DOCX)

S4 File (DOCX)

S5 File (DOCX)

S6 File (XLSX)

S7 File (DOCX)
