# Supplementary material for: Genetically modified food and consumer risk responsibility: The effect of regulatory design and risk type on cognitive information processing
Source: PLoS One. 2021 Jun 9;16(6):e0252580. doi: 10.1371/journal.pone.0252580 (PMC8189520; doi:10.1371/journal.pone.0252580)
Supplement: S1 File — (PDF) [file pone.0252580.s001.pdf]

Appendix I- Registration and data collection
All information provided in this document is translated from Swedish. The Swedish version is available upon request.

Box 1. Welcoming page

You are invited to take part in a study to generate knowledge for Swedish agricultural production.

Researchers at the Swedish University of Agricultural Sciences (SLU) are conducting research on consumer acceptance of novel food products. We seek your participation, which will allow us to learn what people like you would choose among different food products. The insights and knowledge acquired through this study are important for the development of Swedish agriculture and food production. The study will be conducted totally anonymously, and we will not ask any personal information of participants.

The study will be carried out in two steps. First, participants are asked to register here and answer an online questionnaire. Then, participants are required to attend an on-site session to answer some interactive questions (together with other participants a few days after their registration. The study will take place at Uppsala University. Details of the dates and address will be provided after you have completed the registration).

The participants can choose the appropriate time from available options for their on-site session. After completing the study, you will receive a gift card in the amount of 300 SEK for your contribution. You will have a chance to select your gift card from different options, such as cinema tickets or gift cards for grocery stores.

If you are willing to take part in this study, press the bottom below to register. Please note that your identity will be hidden, and we will not use any personal information.

☐ I am above 18 years old.

☐ I am a Swedish citizen.

Security Code: ______

Register Me

Box 2. Consent form

Please read the terms and conditions below carefully. If you would like more information, you can contact Ashkan Pakseresht with ashkan.pakseresht@slu.se.

- This is confirmation that you plan to complete this session and participate in the study taking place in a few days, depending on your availability. You will receive the exact time and location of the venue at the end of the registration. You will also receive a reminder a day before the study.
- The results of this study will be used for purely academic purposes and are strictly confidential.
- The study will be carried out anonymously, with individual's identity and obtained data not being identified to the researchers or other participants.
- The task will involve registering and answering an online questionnaire and in a few days' time, depending on your availability, participating in an on-sight session to answer some interactive questions with other participants.
- Participation in this study is voluntary and you are free to withdraw at any time during the study without giving any reason. In such cases you will not receive a gift card.
- It does not cost you anything to register and withdraw your participation, but we hope you will be able to complete the test.
- The gift cards will be provided at the completion of the study.

☐ I have read the information above and agree with all the conditions.

Agree

Box 3. Demographic questionnaire

Please select your response to the following questions.

What is your income level (gross salary before tax) per month? [▾]

- Please select
- Lower than 20,000 Kr
- Between 20,000 Kr to 40,000 Kr
- More than 40,000 Kr

Please indicate your age. [▾]

What is your educational level? [▾]

What is your gender? [Please select ▾]

How many times do you buy groceries per week? [Please select ▾]

Are you the person in the household that is responsible for grocery purchases? [Please select ▾]

Please insert your E-mail address. The email address will only be used for communication purposes before the study.

Please choose the type of gift card that you would like to receive after completing the experiment. [▾]

Continue

Box 4. Time slots

Please choose a day for the experiment that suits you best from the available options below.
You will receive the address details after you have completed the registration.

Date	Label	
Monday 5th May 2014 (10:00 12:00)	S1C1	
Monday 5th May 2014 (13:30 15:30)	S1C2	◯
Monday 5th May 2014 (16:30 18:30)	S1C3	◯
Tuesday 6th May 2014 (13:30-15:30)	S1C4	◯
Tuesday 6th May 2014 (16:30-18:30)	S2C1	◯
Wednesday 7th May 2014 (10:00-12:00)	S2C2	◯
Wednesday 7th May 2014 (13:30-15:30)	S2C3	◯
Wednesday 7th May 2014 (16:30-18:30)	S2C4	
Thursday 8th May 2014 (13:30-15:30)	S3C1	◯
Friday 9th May 2014 (13:30- 15:30)	S3C2	◯
Friday 9th May 2014 (16:30- 18:30)	S3C3	◯
Saturday 10th May 2014 (13:30-15:30)	S3C4	◯
Saturday 10th May 2014 (16:30-18:30)	S4C1	◯
Monday 12th May 2014 (13:30-15:30)	S4C2	◯
Monday 12th May 2014 (16:30-18:30)	S4C3	◯
Wednesday 14th May 2014 (13:30-15:30)	S4C4	◯
Wednesday 14th May 2014 (16:30-18:30)	S5C1	◯
Thursday 15th May 2014 (13:30-15:30)	S5C2	◯
Thursday 15th May 2014 (16:30-18:30)	S5C3	◯
Friday 16th May 2014 (16:30-18:30)	S5C4	◯

Continue

Box 5. Confirmation[1] of participation.

You are now registered in our system to participate in the experiment.

Your participation code is **1008** and you have selected to participate in the experiment on **Friday 9th May 2014 (16:30- 18:30)**
The experiment will take place at Uppsala University, Blåsenhus. The address is **von Kraemers Allé 1A, 752 37 Uppsala.**

Please remember that from now on we will only use this code to communicate with you and please bring your code to the experiment to log in to the computer. We are looking forward to meeting you soon.

If you have any other questions, you are welcome to contact us for further information.

Click to Print This Page

Frequently asked questions

[1] The information is fictitious and presented for demonstration purposes only.

Appendix II- Questionnaire

Box 1. Welcoming and experiment protocol

Welcome to the experiment - Consumers' Risk Responsibility Judgment in the Context of Genetically Engineered Food.

Thank you for taking the time to participate in our experiment. Your contribution is very important to us!

The aim of the study is to investigate consumers' opinions across different actors in the context of food and their food related choices. The study focuses on interdependent decision analysis from a consumer perspective, meaning that consumer's decision making is part of a broader context where the decision of one player is dependent on the decisions of the other players involved, i.e., farmers, policy makers, retailers, food processing companies. The research is consumer-oriented and is focusing particularly on Swedish consumers.

Please read the following instructions before proceeding to the experiment.

It is important to remember that:

- The responses will be used for purely academic purpose and are strictly confidential.
- You can stop your participation in the experiment whenever you like but you will not receive any compensation if not completing the entire experiment.
- You are not allowed to talk with other participants during the experiment.
- No questions will be answered during the experiment, unless they are regarding technical issues.

The experiment will now start with a short session where you will get the chance practice the type of tasks that are included in the experiment. You need to insert your code and then press the button **"Next"** to continue.

Please enter your code:

Next

Box 2. Scenarios[2]

Please carefully read the scenario profile which will give you a clear picture of the context of the questions that will follow.

<< **Authorities in Sweden have decided to ban application of gene technology in food development and production.**
This scenario means that producing or commercializing Genetically Modified (GM) products, as well as doing research using any type of GM technology within the Swedish borders is not allowed. According to this legislation, growing and importing, as well as processing genetically modified crops and animals or its derivatives for human food or animal feed purposes is banned. Moreover, even research and developments related to the use of gene technology in food production is totally banned**.**
>>

Which of the following aspects are most relevant to you for the evaluation of the above scenario?

Rank 1-4 (where 1=Least relevant, 4=Most relevant)

Environmental aspects

Human health aspects

Socio-economic aspects

Ethical aspects

Next

[2] Four policy scenarios examined in this study include Banned, R&D, Import, Full (described in detail in section 3.2 in the manuscript). Each respondent has been assigned to one of the four scenarios above. The text in the Gray box (texts within angle brackets) is context dependent and describes the scenario in the question. **Box 2 Appendix V** presents an example of 'Banned' scenario in which "authorities in Sweden have decided to ban application of gene technology in food development and production".

Box 3. Eliciting subject's 'Agreement'[3] with risk statements.

Please carefully read the scenario profile which will give you a clear picture of the context of the questions that will follow.

<<**Authorities in Sweden have decided to ban application of gene technology in food development and production**

This scenario means that producing or commercializing Genetically Modified (GM) products, as well as doing research using any type of GM technology within the Swedish borders is not allowed. According to this legislation, growing and importing, as well as processing genetically modified crops and animals or its derivatives for human food or animal feed purposes is banned. Moreover, even research and developments related to the use of gene technology in food production is totally banned**.** >>

According to your evaluation it is obvious that: <<*Ethical aspects*>> associated with GM technology is most concerning you.

Now please indicate to what extent you agree with the statements related to <<*Ethical* >> aspects as below. You can select the answer from the drop-down box.

**Statements**	**Agreement/Disagreement**
By banning the development of GM technology in food production, Swedish society might lose its ability to address global problems, such as the shortage of food, especially in malnourished areas.	Totally disagree<br>Disagree<br>Neither agree nor disagree<br>Agree<br>Totally agree
Banning GM crop cultivation in Sweden, prevents farmers benefiting from increased farm yields. Hence, it might work to decrease farmers income and welfare.	
By banning GM technology, Swedish consumers will have less food alternatives to choose from, so Swedish consumers' freedom of choice might be restricted.	

Next

[3] participants were first asked if they agreed with the relevance of the specific risk statement on a 5-point scale (0 = totally disagree, 0.25 = disagree, 0.5 = neither agree nor disagree, 0.75 = agree, and 1 = totally agree).

Box 4. Eliciting subject's 'Likelihood[4] of risks.

Please carefully read the scenario profile which will give you a clear picture of the context of the questions that will follow.

<<**Authorities in Sweden has decided to ban application of gene technology in food development and production**
This scenario means that producing or commercializing Genetically Modified (GM) products, as well as doing research using any type of GM technology within the Swedish borders is not allowed. According to this legislation, growing and importing, as well as processing genetically modified crops and animals or its derivatives for human food or animal feed purposes is banned. Moreover, even research and developments related to the use of gene technology in food production is totally banned**.**
>>

According to your evaluation it is obvious that: <<*Ethical aspects*>> associated with GM technology is most concerning to you.
In the table below you will see the same statements. Now, please indicate your perception of the likelihood of occurrence of each risk. How likely do you think it is that the risk described in the statements will actually happen? Select the answer from the drop box.

Statements	Likelihood
By banning the development of GM technology in food production, Swedish society might lose its ability to address global problems, such as the shortage of food, especially in malnourished areas.	Not likely at all<br>Very low<br>Low<br>Medium<br>High<br>Almost entirely certain that it will happen
Banning GM crop cultivation in Sweden, prevents farmers benefiting from increased farm yields, hence, it might work to decrease farmers income and welfare.	
By banning GM technology, Swedish consumers will have less food alternatives to choose from, so Swedish consumers' freedom of choice might be restricted.	

Next

[4] Participants were next asked to indicate the perceived likelihood of the respective statement on a 6-point scale (1 = not likely at all to 6 = almost entirely certain that this will happen).

Box 5. Eliciting subject's 'Severity'[5] of risks.

Please carefully read the scenario profile which will give you a clear picture of the context of the questions that will follow.

<<**Authorities in Sweden has decided to ban application of gene technology in food development and production**
This scenario means that producing or commercializing Genetically Modified (GM) products, as well as doing research using any type of GM technology within the Swedish borders is not allowed. According to this legislation, growing and importing, as well as processing genetically modified crops and animals or its derivatives for human food or animal feed purposes is banned. Moreover, even research and developments related to the use of gene technology in food production is totally banned**.** >>

According to your evaluation it is obvious that: <<*Ethical aspects*>> associated with GM technology is most concerning to you.
In the table below you will see the same statements. Now, please indicate your perception of the degree of severity related to the risk of each statement. The level of severity represents how harmful the risk will be to you, should it actually happen? Select the answer from the drop box.

**Statements**	**Severeness**
By banning the development of GM technology in food production, Swedish society might lose its ability to address global problems, such as the shortage of food, especially in malnourished areas.	Not severe at all<br>Somewhat severe<br>Moderately severe<br>Severe<br>Very severe
Banning GM crop cultivation in Sweden, prevents farmers benefiting from increased farm yields. Hence, it might work to decrease farmers income and welfare.	
By banning GM technology, Swedish consumers will have less food alternatives to choose from, so Swedish consumers' freedom of choice might be restricted.	

5 Participants were then asked to rate the severity of the event, if it was to occur, on a 5-point scale (1 = not severe at all to 5 = very severe).

Box 6. Eliciting subject's 'Controllability[6] of risks.

Please carefully read the scenario profile which will give you a clear picture of the context of the questions that will follow.

<<**Authorities in Sweden has decided to ban application of gene technology in food development and production**
This scenario means that producing or commercializing Genetically Modified (GM) products, as well as doing research using any type of GM technology within the Swedish borders is not allowed. According to this legislation, growing and importing, as well as processing genetically modified crops and animals or its derivatives for human food or animal feed purposes is banned. Moreover, even research and developments related to the use of gene technology in food production is totally banned. >>

According to your evaluation it is obvious that: <<*Ethical aspects*>> associated with GM technology is most concerning to you.
In the table below you will see the same statements. Now, please indicate your ability to control the risks associated with each statement. Controllability represents your ability to command the situation in which you are exposed to each risk. (Can you avoid the risk or prepare yourself by taking preventive measures to avoid it or reduce its impact on you?). Select the answer from the drop box.

Statements	Control
By banning the development of GM technology in food production, Swedish society might lose its ability to address global problems such as the shortage of food, especially in malnourished areas.	I can completely control my exposure to this risk<br>I can to great degree control my exposure to this risk<br>I can to some extent control my exposure to this risk<br>I can (only) to a small degree control my exposure to this risk<br>I have no control at all over my exposure to this risk
Banning GM crop cultivation in Sweden, prevents farmers benefiting from increased farm yields, hence, it might work to decrease farmers income and welfare.	
By banning GM technology, Swedish consumers will have less food alternatives to choose from, so Swedish consumers' freedom of choice might be restricted.	

Next

[6] Participants were then asked to rate the controllability of the event, if it were to occur, on a 5-point scale (1 = I have no control at all over my exposure to this risk, 5 = I can completely control my exposure to this risk).

Box 7. Overall judgement[7] of risks.

In previous pages you have indicated your evaluation regarding the degree of severity, the likelihood of occurrence, and controllability associated with each statement as:

Statements	Severeness	Likelihood	Control
By banning the development of GM technology in food production, Swedish society might lose its ability to address global problems, such as the shortage of food, especially in malnourished areas.	*Somewhat severe*	*Not likely at all*	*I can (only) to a small degree control my exposure to this risk*
Banning GM crop cultivation in Sweden, prevents farmers benefiting from increased farm yields. Hence, it might work to decrease farmers income and welfare.	*Somewhat severe*	*Low*	*I can to some extent control my exposure to this risk*
By banning GM technology, Swedish consumers will have less food alternatives to choose from, so Swedish consumers' freedom of choice might be restricted.	*Moderately severe*	*Not likely at all*	*I can completely control my exposure to this risk*

We believe that you have used your best judgment to evaluate these statements, however we would like to ask you how confident you feel about the evaluation of likelihood, severity, and controllability that you have given above. Select the answer from the drop box.

Not confident at all
To small degree confident
To some degree confident
To a great degree confident
Completely confident

Next

[7] The response values in this box are just for demo purpose.

Box 8. Eliciting subject's 'Controllability'[8] of risks.

You have just provided your answers to statements regarding Ethical aspects of risks associated with GM food. Please, use the table below to allocate your view on the share of responsibility that each decision maker in the food chain should have. There is nobody else other than you who will know the distribution of responsibility you give. Think of yourself when filling in the extent to which consumers are responsible.
Attribute values between 0-100% (where 0= No responsibility, 100= Full responsibility).

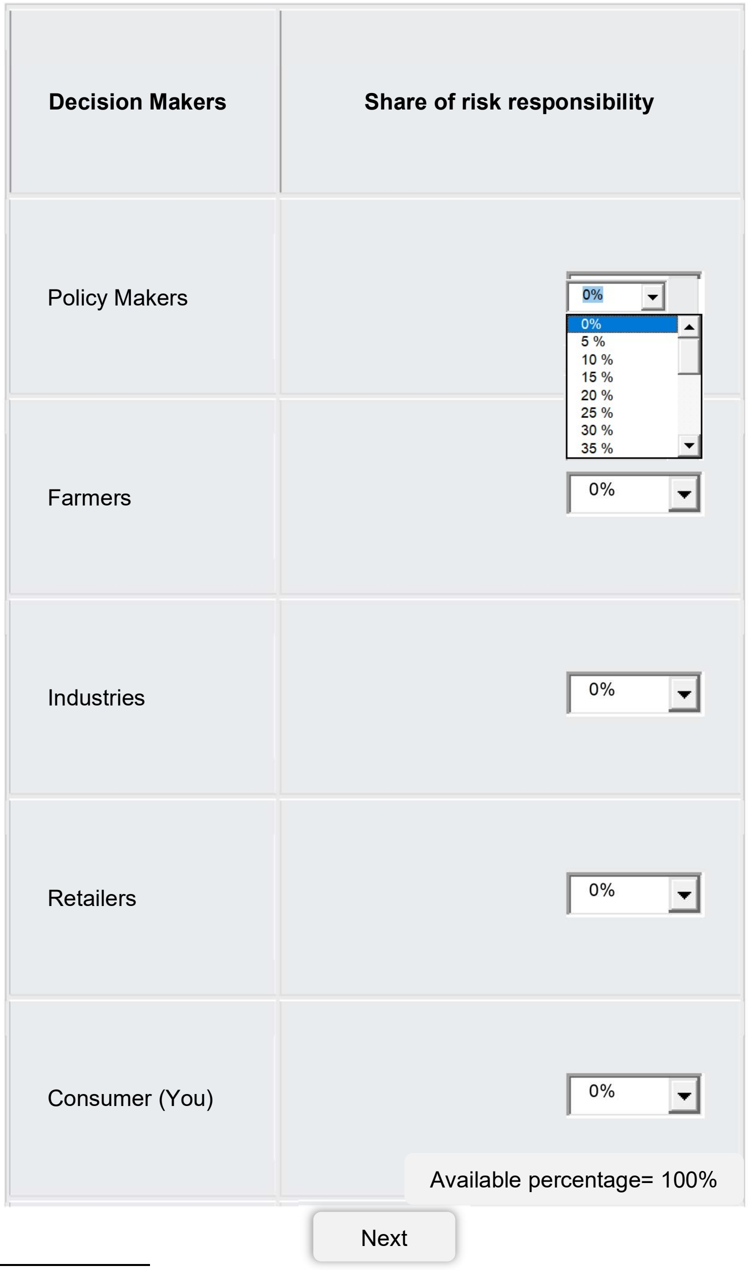

[8] Participants were given 100 marks to distribute across food value chain actors, to indicate the share of responsibility given to each actor type. Participants could adjust and verify the distribution of their marks before completing the task.
