# Supplementary material for: Genetically modified food and consumer risk responsibility: The effect of regulatory design and risk type on cognitive information processing
Source: PLoS One. 2021 Jun 9;16(6):e0252580. doi: 10.1371/journal.pone.0252580 (PMC8189520; doi:10.1371/journal.pone.0252580)
Supplement: S2 File — (DOCX) [file pone.0252580.s002.docx]

***Appendix III.* Material presented to the participents**

Table C1. Description of four scenarios randomly presented to the participents (for review purposes only)

| **Policy Scenario** | **Description** |
| --- | --- |
| **Banned** | **Authorities in Sweden has decided to ban application of gene technology in food development and production.**  This scenario means that producing or commercializing Genetically Modified (GM) products, as well as doing research using any type of GM technology within the Swedish borders is not allowed. According to this legislation, growing and importing, as well as processing genetically modified crops and animals or its derivatives for human food or animal feed purposes is banned. Moreover, even research and developments related to the use of gene technology in food production is totally banned**.** |
| **Research and Development (R&D)** | **Only research on Genetically Modified food is allowed by Swedish authorities.**  This scenario means that, under certain regulations, only research and development (R&D) using Genetically Modified (GM) technology are allowed, i.e., in laboratories or control filed trials within the Swedish borders. According to this scenario, producing and commercializing GM products is not allowed in Sweden. |
| **Import with mandatory labelling** | **Only importation of Genetically Modified Food is allowed.**  This scenario means that it is only allowed to import Genetically Modified (GM) products to Sweden after the required food safety regulations are met. In other words, farmers are not allowed to grow GM crops, but the food industry is allowed to import GM food under certain regulations and with mandatory labelling for commercialization. |
| **Full commercialization with mandatory labelling** | **Authorities allow commercialization and cultivation of Genetically Modified Food.**  This scenario means that production and commercialization of Genetically Modified (GM) food is permitted in Sweden after the required food safety regulation are met. The GM foods can be commercialized either under public owned patent rights or private patent rights with mandatory labelling. In other words, farmers are allowed to produce GM foods domestically and the food industry is allowed to import GM foods produced in other parts of the world. |

**STRUCTURE OF THE FOOD CHAIN**

**Regulatory bodies**

- Provide policies, regulations, and legislations that have to be respected by everyone on the food chain.
- Includes national and international rules and regulations, i.e., EU legislations for food production and sale, including food quality and food safety.

**Swedish Farmers**

- Grow crops and/or manage livestock production.

**Food Industry**

- Deal with the manufacturing and processing/preparation of food products.

**Retailers** (such as ICA, Coop and Willy’s)

- Purchase goods or products from the food industry and sell to consumers.

**Consumers (You)**

- The final buyer of the products.


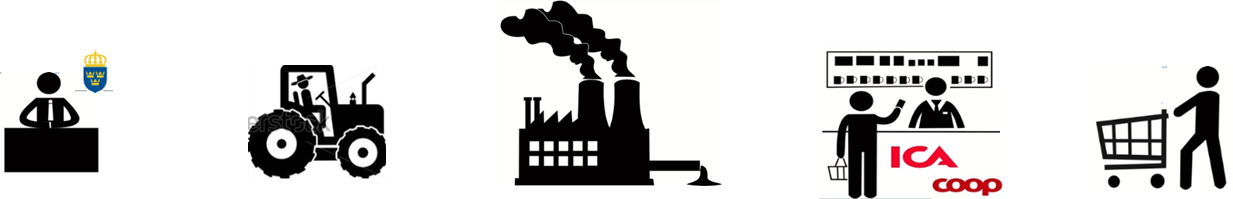


*Fig. C2. Structure and main role of the food value chain (FVD) actors.* *(for review purposes only)*

***Appendix IV*. Demographic sample characteristics**

***Table D1****.* Demographic sample characteristics (shares) by policy scenario compared with Swedish population statistics.

| **Variable** | **Banned** |  | **R&D** |  | **Import** |  | **Full** |  | **Sweden** |
| --- | --- | --- | --- | --- | --- | --- | --- | --- | --- |
| ***Gender*** *(=1 if male****)*** | 0.40 |  | 0.41 |  | 0.41 |  | 0.48 |  | 0.5 |
| ***Age^a^*** |  |  |  |  |  |  |  |  |  |
| 18-35 years old | 0.38 |  | 0.45 |  | 0.37 |  | 0.36 |  | 0.33 |
| 36-60 years old | 0.34 |  | 0.30 |  | 0.34 |  | 0.34 |  | 0.44 |
| 61-75 years old | 0.28 |  | 0.25 |  | 0.29 |  | 0.30 |  | 0.23 |
| ***Level of highest education*** |  |  |  |  |  |  |  |  |  |
| Primary or below | 0.04 |  | 0.04 |  | 0.02 |  | 0.04 |  | 0.17 |
| Secondary school | 0.28 |  | 0.25 |  | 0.28 |  | 0.27 |  | 0.45 |
| College or equiv. (<3 years) | 0.19 |  | 0.21 |  | 0.34 |  | 0.29 |  | 0.14 |
| University or equiv. (>3 years) | 0.49 |  | 0.50 |  | 0.36 |  | 0.40 |  | 0.21 |
| No information | 0 |  | 0 |  | 0 |  | 0 |  | 0.02 |
| ***Household income^b,c^*** |  |  |  |  |  |  |  |  |  |
| <20,000 SEK | 0.43 |  | 0.48 |  | 0.49 |  | 0.44 |  | N.A. |
| 20,000-40,000 SEK | 0.49 |  | 0.42 |  | 0.38 |  | 0.46 |  | N.A. |
| > 40,000 SEK | 0.08 |  | 0.10 |  | 0.13 |  | 0.10 |  | N.A. |
| Total sample size | 129 |  | 134 |  | 137 |  | 135 |  |  |

^a^ According to Statistics Sweden, the Swedish population between 18 to 75 years old on Dec. 31, 2014 was 7,004,264.

^b^ Gross monthly.

^c^ There are no available official statistics on gross income per household. However, according to Statistics Sweden, the average monthly gross income for 2013 per age class was: 15,914 SEK (age 18-35); 28,694 SEK (age 36-60); and 22,370 SEK (age 61-75).

*Appendix V.* Summary Statistics from Moderated-Mediation Analysis

*Table E1*. Unstandardized OLS regression coefficients related to health risk dimension with confidence intervals (standard Errors in parenthesis), estimating perceived risk, self-control, and attribution of risk responsibility to self.

|  | Perceived  Risk (*PR*) | | |  | Perceived  Self-Control (*SC*) | | |  | Self-Risk  Responsibility (*RR*) | | |
| --- | --- | --- | --- | --- | --- | --- | --- | --- | --- | --- | --- |
|  |  | Coeff. | 97.5% *CI* |  |  | Coeff. | 97.5% *CI* |  |  | Coeff. | 97.5% *CI* |
| R&D (*PS*_1_) | $a_{11}$_→_ | 0.55^***^ | 0.271, 0.828 |  | $a_{21}$_→_ | -0.153^*^ | -0.351, 0.045 |  | $\acute{c}_{1}$_→_ | -1.07 | -0.403, 0.190 |
| Scenario |  | (0.124) |  |  |  | (0.088) |  |  |  | (0.132) |  |
| Import (*PS*_2_) | $a_{12}$_→_ | 1.01^***^ | 0.732, 1.293 |  | $a_{22}$_→_ | -0.212^**^ | -0.429, -0.005 |  | $\acute{c}_{2}$_→_ | 0.20 | -0.126, 0.526 |
| Scenario |  | (0.125) |  |  |  | (0.096) |  |  |  | (0.145) |  |
| Full (*PS*_3_) | $a_{13}$_→_ | 0.97^***^ | 0.693, 1.251 |  | $a_{23}$_→_ | -0.227^**^ | -0.440, -0.013 |  | $\acute{c}_{3}$_→_ | 0.051 | -0.271, 0.373 |
| Scenario |  | (0.124) |  |  |  | (0.095) |  |  |  | (0.143) |  |
| Perceived (*PR*) |  |  |  |  | *d* _→_ | 0.298^***^ | 0.198, 0.397 |  | $b_{1}$_→_ | -0.123^*^ | -0.285, 0.038 |
| Risk |  |  |  |  |  | (0.044) |  |  |  | (0.072) |  |
| Perceived (*SC*) |  |  |  |  |  |  |  |  | $b_{2}$_→_ | 0.23^**^ | 0.143, 0.449 |
| Self-Control |  |  |  |  |  |  |  |  |  | (0.096) |  |
| Gender (*U*_1_) | $\gamma_{11}$_→_ | -0.073 | -0.271, 0.126 |  | $\gamma_{12}$_→_ | 0.076 | -0.060, 0.212 |  | $\theta_{1}$_→_ | -0.105 | -0.308, 0.098 |
|  |  | (0.088) |  |  |  | (0.060) |  |  |  | (0.900) |  |
| Age_1_ (*U*_21_) | $\gamma_{211}$_→_ | 0.210^*^ | -0.058, 0.478 |  | $\gamma_{212}$_→_ | -0.48 | -0.232, 0.136 |  | $\theta_{21}$_→_ | -0.167 | -0.442, 0.108 |
|  |  | (0.119) |  |  |  | (0.082) |  |  |  | (0.122) |  |
| Age_2_ (*U*_22_) | $\gamma_{221}$_→_ | 0.163 | -0.097, 0.422 |  | $\gamma_{222}$_→_ | -0.145^*^ | -0.323, 0.034 |  | $\theta_{22}$_→_ | -0.296^**^ | -0.563, -0.029 |
|  |  | (0.115) |  |  |  | (0.079) |  |  |  | (0.119) |  |
| Educa_1_ (*U*_31_) | $\gamma_{311}$_→_ | -0.154 | -0.619, 0.310 |  | $\gamma_{312}$_→_ | -0.154 | -0.471, 0.164 |  | $\theta_{31}$_→_ | -0.184 | -0.658, 0.290 |
|  |  | (0.206) |  |  |  | (0.141) |  |  |  | (0.210) |  |
| Educa_2_ (*U*_32_) | $\gamma_{321}$_→_ | -0.232 | -0.696, 0.231 |  | $\gamma_{322}$_→_ | -0.145 | -0.462, 0.172 |  | $\theta_{32}$_→_ | -0.085 | -0.559, 0.389 |
|  |  | (0.205) |  |  |  | (0.141) |  |  |  | (0.210) |  |
| Educa_3_ (*U*_33_) | $\gamma_{331}$_→_ | -0.254 | -0.708, 0.200 |  | $\gamma_{332}$_→_ | -0.153 | -0.464, 0.158 |  | $\theta_{33}$_→_ | -0.086 | -0.551, 0.379 |
|  |  | (0.201) |  |  |  | (0.138) |  |  |  | (0.206) |  |
| Income_1_ (*U*_41_) | $\gamma_{411}$_→_ | 0.022 | -0.212, 0.255 |  | $\gamma_{412}$_→_ | 0.040 | -0.119, 0.200 |  | $\theta_{41}$*→* | 0.081 | -0.157, 0.319 |
|  |  | (0.104) |  |  |  | (0.071) |  |  |  | (0.105) |  |
| Income_2_ (*U*_42_) | $\gamma_{421}$_→_ | 0.217 | -0.140, 0.573 |  | $\gamma_{422}$_→_ | 0.150 | -0.063, 0.363 |  | $\theta_{42}$*→* | 0.267^*^ | -0.099, 0.632 |
|  |  | (0.158) |  |  |  | (0.108) |  |  |  | (0.162) |  |
| Constant | $i_{PR}$_→_ | 1.398^***^ | 0.902, 1.894 |  | $i_{SC}$_→_ | 0.051 | -0.094, 0.394 |  | $i_{RR}$_→_ | 2.798^***^ | 2.252, 3.344 |
|  |  | (0.220) |  |  |  | (0.162) |  |  |  | (0.242) |  |
|  | $R^{2}$=0.2852 | | |  | $R^{2}$ = 0.1940 | | |  | $R^{2}$=0.0646 | | |
|  | *F*(11, 240) = 8.704 , *P* < .01 | | |  | *F*(12, 239) = 4.794 , *P* < .001 | | |  | *F*(11, 240) = 1.508 , *P* > 0.1 | | |
| ^*^*P* < .1, ^**^*P* < .05, ^***^*P* < .01.  Dummy variables PS_1_, PS_2_, and PS_3_ represent multicategory independent variables, i.e., policy regimes which banned scenario serves as reference group.  Dummy variable U_1_ represents “gender” in which U_1_=male (reference group: female).  Dummy variables U_21_ and U_22_ represent “age” which U_21_ = 36<age<60, U_22_ = 60<age<75 (reference group: age 18-35).  Dummy variables U_31_, U_32_ and U_33_ represent variable “education level” that U_31_ = upper secondary, U_32_ = undergraduate level, U_33_ = postgraduate level (reference group: primary school).  Dummy U_41_ and U_42_ represent variable “income” where U_41_ = moderate, U_42_ = high (reference group: low income).  R^2^ is the squared multiple correlation.  Estimations are derived from PROCESS macros for SPSS (Hayes & Preacher, 2014). | | | | | | | | | | | |
