# Supplementary material for: Genetically modified food and consumer risk responsibility: The effect of regulatory design and risk type on cognitive information processing
Source: PLoS One. 2021 Jun 9;16(6):e0252580. doi: 10.1371/journal.pone.0252580 (PMC8189520; doi:10.1371/journal.pone.0252580)
Supplement: S3 File — (DOCX) [file pone.0252580.s003.docx]

**Appendix VI. Distribution of Risk Responsibility (RR)** **across treatments and risk dimensions.**


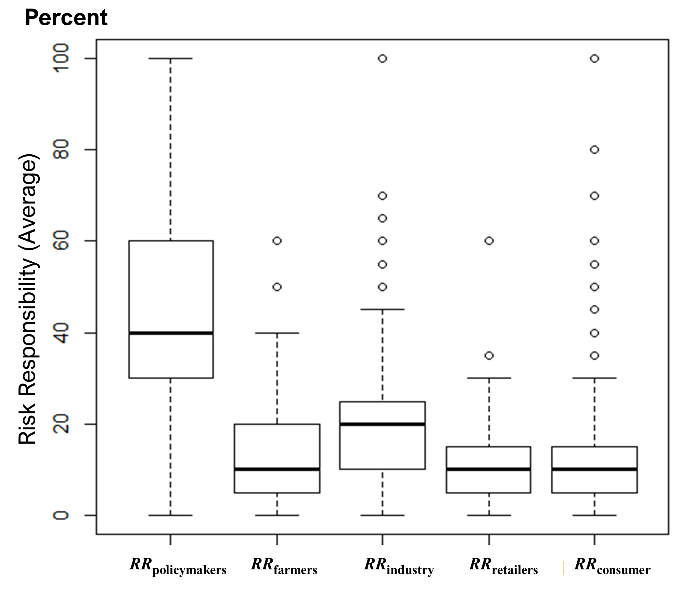


**Figure F1**. Boxplots representing distribution of overall Risk Responsibility (RR) attributed (constant sum scaling of 100 percent) to different value chain actors (i.e., Policymakers, Farmers, Industry, Retailers) and to self (as consumer).

**Box F2. Friedman’s test:**

H0: Attribution of RR across food chain actors is not significantly different.

H1: Attribution of RR across food chain actors is significantly different.

The test statistic for the Friedman’s test is a chi-square with [(number of actors)-1] degrees of freedom. Friedman chi-squared = 909.58, df= 4, p-value < 0.001

The p-value (p-value < 0.001) for this test is very small. It is therefore plausible that the RR distribution across actor groups have statistically significant different medians.

Post hoc analysis**:**

Pairwise comparisons using Wilcoxon signed rank test. The p-value are adjusted using Holm (1979) method for multiple comparisons.

Res_P Res_F Res_I Res_R

Res_F 2e-16 - - -

Res_I 2e-16 8.3e-16 - -

Res_R 2e-16 3.6e-12 2e-16 -

Res_C 2e-16 1.1e-05 2e-16 0.08

Hence, only consumers and retailers are judged as having a similar distribution of responsibility shares.

**Table F3**. Mean of Risk Responsibility percentages attributed to food chain actors (i.e., Policymakers, Industry, Farmers, and Retailers) and to self (consumers).

| ***Risk Responsibility (RR)*** | **Banned** | **R&D** | **Import** | **Full** | **Average** |
| --- | --- | --- | --- | --- | --- |
| *RR*_policymakers_ | 46.7 | 48.5 | 44.5 | 41.9 | **45.4** |
| *RR*_farmers_ | 14.0 | 14.9 | 10.7 | 15.4 | **13.7** |
| *RR*_industry_ | 18.1 | 16.9 | 22.2 | 21.1 | **19.6** |
| *RR*_retailers_ | 9.1 | 9.6 | 10.4 | 10.4 | **9.9** |
| *RR*_consumers_ | 12.1 | 10.1 | 12.2 | 11.2 | **11.4** |
| Total | *100.0* | *100.0* | *100.0* | *100.0* | *100.0* |

**Distribution of Self Risk Responsibility (SRR) across scenarios and risk dimensions**


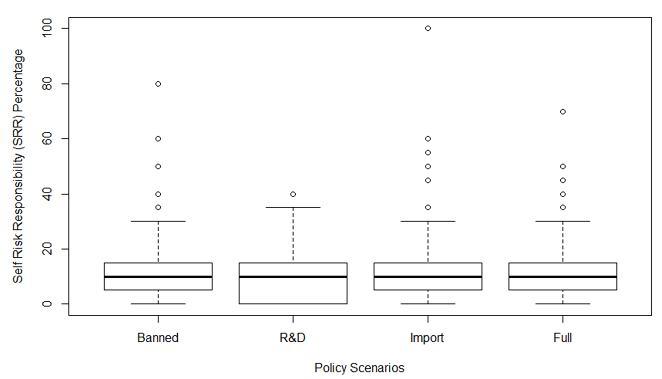

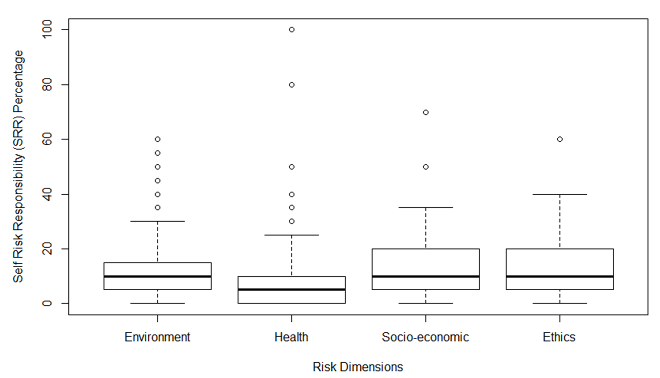


**Figure F4**. Box plots illustrating distribution of Self Risk Responsibility (SRR) across risk dimensions (left) and scenarios (right).

**Box F5. Kruskal-Wallis test:**

H0: There is no significant difference in the distribution of SRR across policy scenarios.

H1: There is a significant difference in the distribution of SRR across policy scenarios.

Kruskal-Wallis rank sum test results did not reject the null hypothesis:

Kruskal-Wallis chi-squared = 2.0032, df = 3, p-value = 0.5717

H0: There is no significant difference in the distribution of SRR between risk dimensions.

H1: There is a significant difference in the distribution of SRR between risk dimensions.

Kruskal-Wallis rank sum test results rejected the null hypothesis:

Kruskal-Wallis chi-squared = 21.399, df = 3, p-value = 8.697e-05

Comparison of x by group

(Bonferroni)

Col Mean-|

Row Mean | Environment Ethics Health

---------+---------------------------------

Ethics | -0.241699

| 1.0000

|

Health | 3.263516 2.852371

| 0.0033* 0.0130*

|

Socio-ec | -1.142835 -0.810843 -3.628736

| 0.7593 1.0000 0.0009*

alpha = 0.05

Reject Ho if p <= alpha/2

Hence distribution of SRR is significantly different across risk dimensions, meaning concerns for GM food in terms of human health risks, provoked different shares of self-risk responsibility attribution.

**Table F6**. Average self-risk responsibility (SRR) percentages and number of participants (N) in each policy scenario-risk type dimension.

|  |  | **Policy scenario** | | | |  |  |  |
| --- | --- | --- | --- | --- | --- | --- | --- | --- |
| **Risk Dimension** |  | Banned | R&D | Import | Full |  | Total |  |
| Environmental |  | 12.76  (35) | 10.0  (34) | 14.72  (36) | 12.36  (36) |  | 12.47  (141) |  |
| Health |  | 10.19  (52) | 7.57  (68) | 11.79  (66) | 9.09  (66) |  | 9.66  (252) |  |
| Socio-economic |  | 13.33  (12) | 16.77  (16) | 10.59  (17) | 17.50  (16) |  | 14.55  (61) |  |
| Ethical |  | 14.62  (25) | 14.33  (15) | 9.71  (17) | 10.88  (17) |  | 12.38  (74) |  |
| Total |  | 12.72  (124) | 12.17  (133) | 11.7  (136) | 12.46  (135) |  | 12.26  (528) |  |
