# Supplementary material for: Genetically modified food and consumer risk responsibility: The effect of regulatory design and risk type on cognitive information processing
Source: PLoS One. 2021 Jun 9;16(6):e0252580. doi: 10.1371/journal.pone.0252580 (PMC8189520; doi:10.1371/journal.pone.0252580)
Supplement: S4 File — (DOCX) [file pone.0252580.s004.docx]

**Appendix VIII**. Distribution of self-control perception across treatments and risk dimensions.


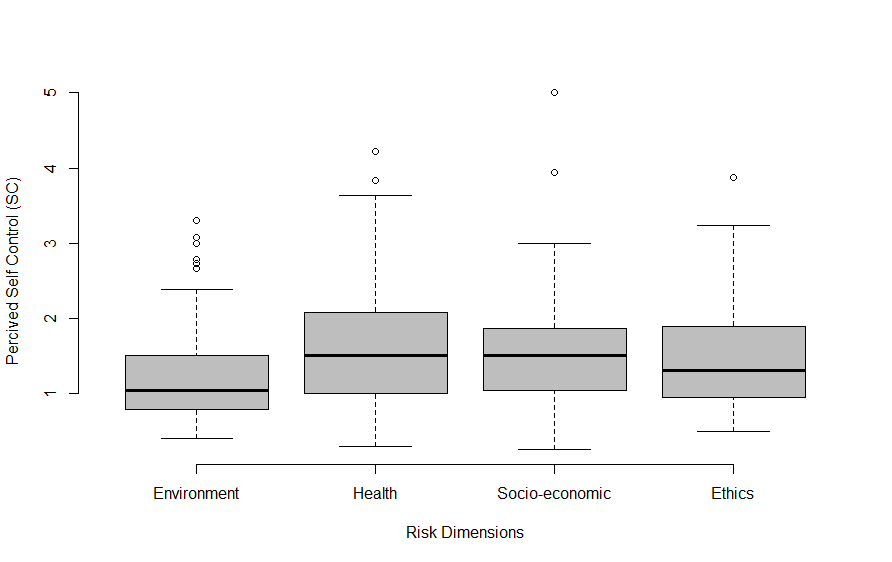


**Figure G1**. Boxplot distribution of self-control (SC) over risk dimensions (Mean SC_Env_= 1.2, SC_Health_= 1.6, SC_Socio_= 1.6, SC_Ethics_= 1.5). Fligner-Killeen test rejected the homogeneity of variances across risk dimensions (chi-squared = 11.356, df = 3, p-value = 0.009). Kruskal-Wallis rank sum test performed and indicated statistically significant difference of SC mean across risk dimensions (Kruskal-Wallis chi-squared = 27.87, df = 3, p-value < 0.001). Post hoc analysis (Bonferroni P-value adjusted= 0.025) confirmed significant differences between mean SC environmental-health and environmental-socio-economic risk dimensions.

**Box G2. Kruskal-Wallis rank sum test (SC across Risk Dimensions):**

H0: Self-control perception across risk dimensions is not significantly different.

H1: Self-control perception across risk dimensions is significantly different.

Fligner-Killeen test rejected the homogeneity of variances across risk dimensions (chi-squared = 11.356, df = 3, p-value = 0.009).

Kruskal-Wallis rank sum test were performed and indicated statistically significant difference of SC mean across risk dimensions (Kruskal-Wallis chi-squared = 27.87, df = 3, p-value < 0.001).

Post hoc analysis**:**

Post hoc Dunn test (Bonferroni P-value adjusted= 0.025) confirmed significant differences between mean SC environmental-health and environmental-socio-economic risk dimensions.

Col Mean-|

Row Mean | Environm Ethics Health

---------+---------------------------------

Ethics | -2.598051

| 0.0281

|

Health | -5.133551 -1.262655

| 0.0000* 0.6201

|

Socio-ec | -3.365597 -0.825948 0.168963

| 0.0023* 1.0000 1.0000

alpha = 0.05

Reject Ho if p <= alpha/2


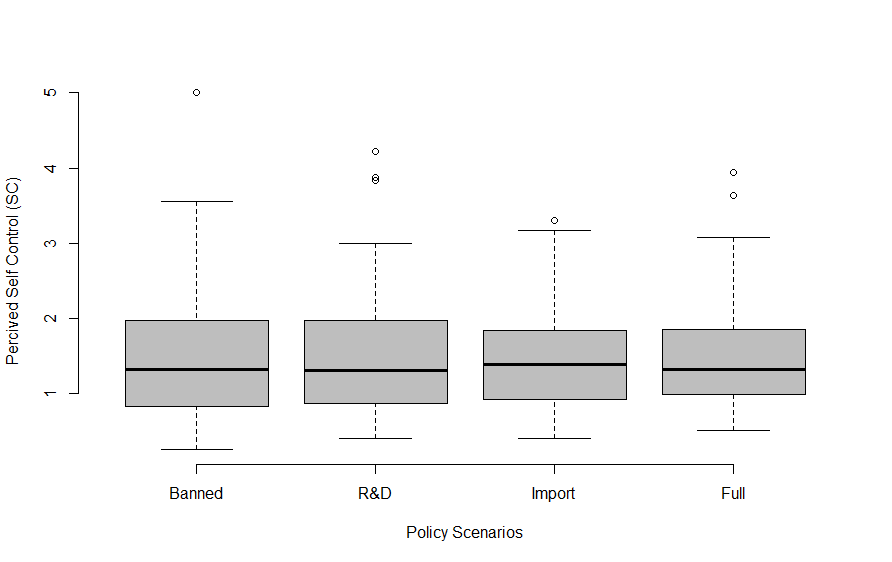


**Figure G3**. Box plots mean SC across policy scenarios (Mean SC_Banned_= 1.45, SC_R&D_= 1.48, SC_Import_= 1.46, SC_Full_= 1.47). There were no statistically significant differences between group means as determined by one-way ANOVA (F(3,524) = 0.03, p = .99).

**Box G4. Kruskal-Wallis rank sum test (SC across Policy Scenarios):**

H0: Self-control perception across policy scenarios is not significantly different.

H1: Self-control perception across policy scenarios is significantly different.

Fligner-Killeen test did not reject the homogeneity of variances across scenarios (chi-squared = 6.7709, df = 3, p-value = 0.07).

Fligner-Killeen test of homogeneity of variances

data: SC by scenario

Fligner-Killeen:med chi-squared = 6.7709, df = 3, p-value = 0.07957

Then, ANOVA test was performed and indicated no statistically significant difference of SC mean across scenarios.

Df Sum Sq Mean Sq F value Pr(>F)

newdata$scenario 3 0.05 0.0152 0.03 0.993

Residuals 524 268.77 0.5129

TukeyHSD

Tukey multiple comparisons of means

95% family-wise confidence level


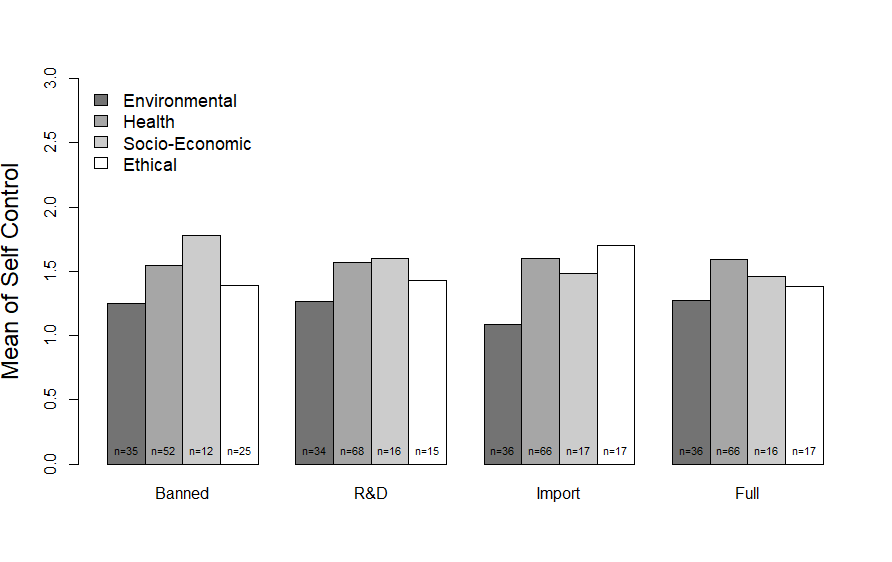


**Figure G5**. Mean Self-Control (SC) perception by risk dimensions and across scenarios. Kruskal-Wallis tests indicated only for those participated in the import scenario, SC is differently perceived between environmental risks and health risks, as well as ethical risks. types ($X_{Banned}^{2}$= 4.0, df= 3, P-value=0.3; $X_{R\&D}^{2}$= 5.2, df= 3, P-value=0.2; $X_{Import}^{2}$= 19.6, df= 3, P-value=0.0002; $X_{Full}^{2}$= 5.7, df= 3; P-value=0.1).

**Box G6. Kruskal-Wallis test separated data based on policy scenario:**

H0: Self-control perception divided by policy scenarios is not significantly different with respect to risk dimensions.

H1: Self-control perception divided by policy scenarios is significantly different with respect to risk dimensions.

**- Kruskal-Wallis rank sum test (people in Banned scenario):**

data: Self-control perception for participants in **Banned** scenario by risk dimension

Kruskal-Wallis chi-squared = 4.0143, df = 3, p-value = 0.299

**- Kruskal-Wallis rank sum test (people in R&D scenario):**

data: Self-control perception for participants in **R&D** scenario by risk dimension

Kruskal-Wallis chi-squared = 5.1695, df = 3, p-value = 0.1598

**- Kruskal-Wallis rank sum test (people in Import scenario):**

data: Self-control perception for participants in **Import** scenario by risk dimension

Kruskal-Wallis chi-squared = 19.556, df = 3, p-value = 0.0002097*

data: x and group

Kruskal-Wallis chi-squared = 19.5563, df = 3, p-value = 0

Comparison of SC (in Import) by group

(Bonferroni)

Col Mean-|

Row Mean | Environm Ethics Health

---------+---------------------------------

Ethics | -3.294059

| 0.0030*

|

Health | -4.082893 0.453809

| 0.0001* 1.0000

|

Socio-ec | -2.540479 0.646548 0.361550

| 0.0332 1.0000 1.0000

alpha = 0.05; Reject Ho if p <= alpha/2

**- Kruskal-Wallis rank sum test (people in Full scenario):**

data: Self-control perception for participants in **Full** scenario by risk dimension

Kruskal-Wallis chi-squared = 5.6526, df = 3, p-value = 0.1298


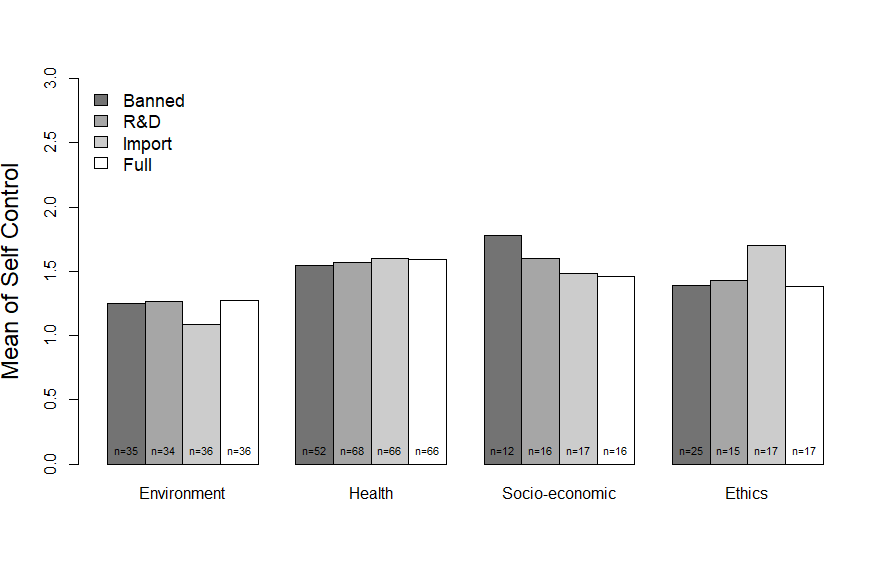


**Figure G7**. Distribution of Self-control (SC) separated based on risk type and across policy scenarios. Kruskal-Wallis tests indicated there is not association between policy treatments on SC respect to each of the risk types ($X_{Env}^{2}$= 4.2, df= 3, P-value=0.2; $X_{Health}^{2}$= 0.66, df= 3, P-value=0.88; $X_{socio}^{2}$= 1.14, df= 3, P-value=0.77; $X_{Ethics}^{2}$= 3.6, df= 3, P-value=0.3).

**Box G8. Kruskal-Wallis test separated data based on Risk Dimensions:**

H0: Self-control perception divided by risk dimension is not significantly different with respect to scenarios.

H1: Self-control perception divided by risk dimension is significantly different with respect to scenarios.

**- Kruskal-Wallis rank sum test (Environmental risk dimension):**

data: Self-control perception for participants with Environmental concerns

across policy scenarios.

Kruskal-Wallis chi-squared = 4.1625, df = 3, p-value = 0.2444

**- Kruskal-Wallis rank sum test (Health risk dimension):**

data: Self-control perception for participants with **health risks** across

policy scenarios.

Kruskal-Wallis chi-squared = 0.6567, df = 3, p-value = 0.8833

**- Kruskal-Wallis rank sum test (Socio-economic risk dimension):**

data: Self-control perception for participants with **socio-economic risks** across

policy scenarios.

Kruskal-Wallis chi-squared = 1.137, df = 3, p-value = 0.7681

**- Kruskal-Wallis rank sum test (Ethical risk dimension):**

data: Self-control perception for participants with **Ethical concerns** across

policy scenarios.

Kruskal-Wallis chi-squared = 3.582, df = 3, p-value = 0.3103
