# Supplementary material for: Genetically modified food and consumer risk responsibility: The effect of regulatory design and risk type on cognitive information processing
Source: PLoS One. 2021 Jun 9;16(6):e0252580. doi: 10.1371/journal.pone.0252580 (PMC8189520; doi:10.1371/journal.pone.0252580)
Supplement: S5 File — (DOCX) [file pone.0252580.s005.docx]

***Appendix VII***. Distribution of Perceived Risk perception across treatments and risk dimensions.


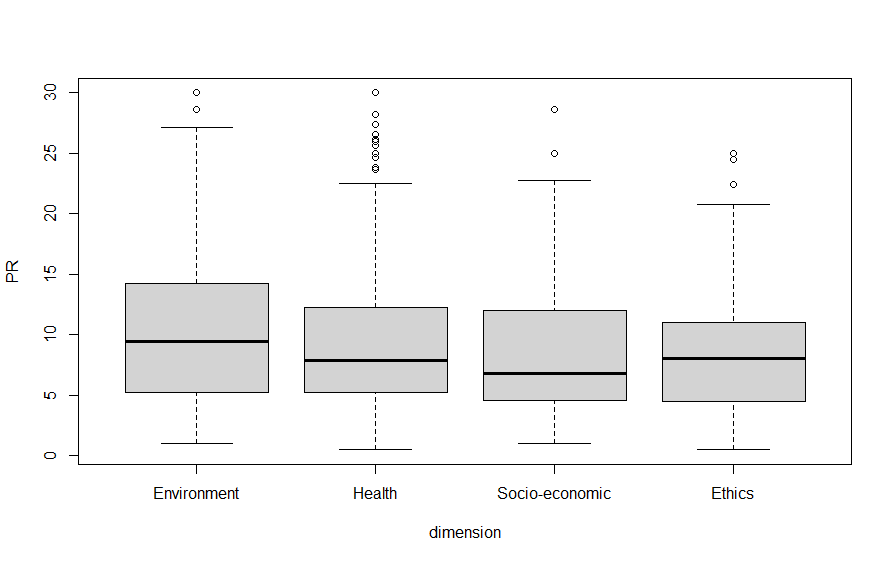


**Figure H1**. Boxplots comparing distribution of Perceived Risk (PR) across risk dimensions.


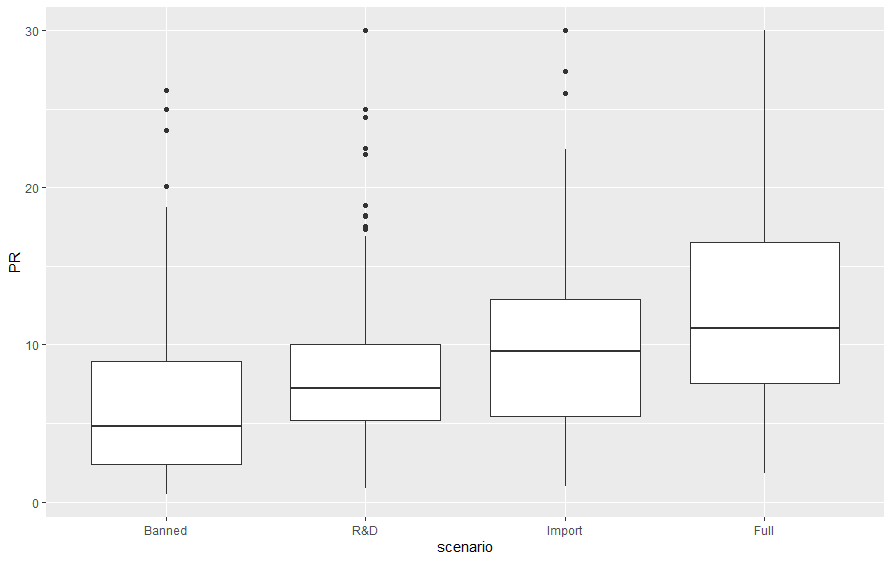


**Figure H2**. Box plot showing distribution of Perceived Risk (PR) across scenarios.


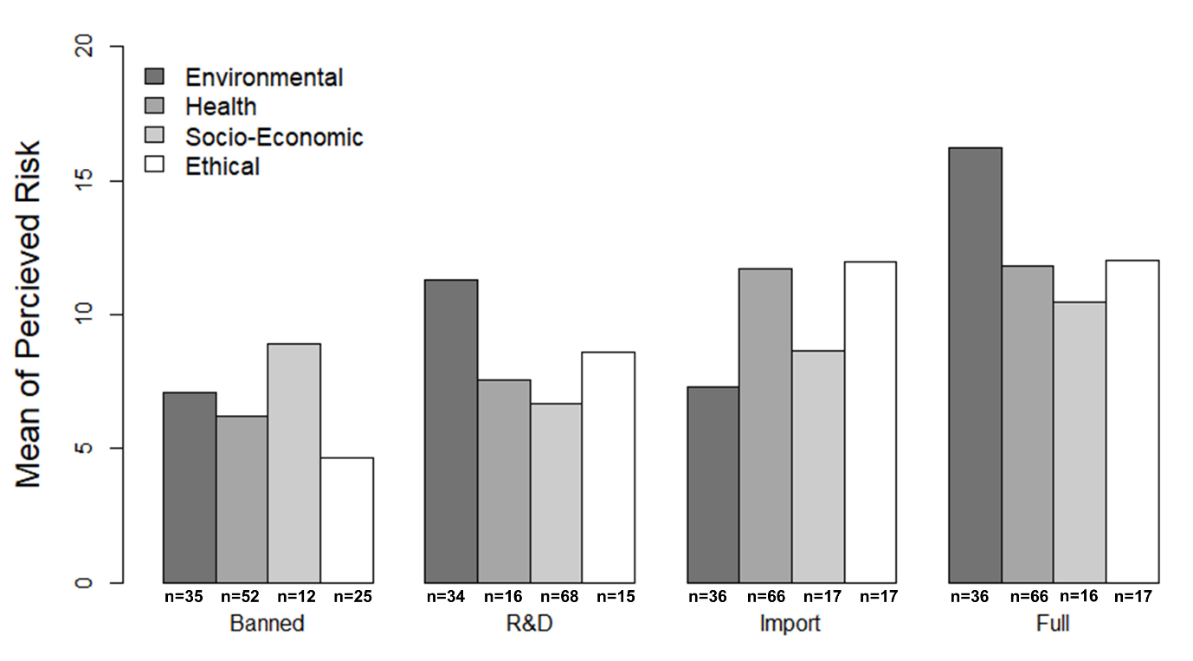


**Figure H3**. Mean Perceived Risk across scenarios and risk dimensions (PR_min_= 0.5, PR_max_=30, PR_mean_= 9.56, PR_median_= 8.18).

**Table H4**. chi-squared Kruskal-Wallis (KW) and Fligner-Killeen (FK) test statistics of pairwise comparisons of Perceived Risk (PR) across scenarios and separated by risk dimensions.

|  |  | **Environmental** |  | **Health** |  | **Socio-Economic** |  | **Ethical** |
| --- | --- | --- | --- | --- | --- | --- | --- | --- |
| **Multiple comparison** **between treatments** |  | ${}_{FK}^{2}$= 10.9^**^**^a^** |  | ${}_{FK}^{2}$= 16.4^***^ |  | ${}_{FK}^{2}$= 11.8^***^ |  | ${}_{FK}^{2}$= 7.2 |
|  |  | ${}_{KW}^{2}$= 43.6^***^**^b^** |  | ${}_{KW}^{2}$= 53.4^***^ |  | ${}_{KW}^{2}$= 3.5 |  | ${}_{KW}^{2}$= 27.9^***^ |
|  |  | ${}_{\boldsymbol{KW, paired}}^{\boldsymbol{2}}$c |  | ${}_{\boldsymbol{KW, paired}}^{\boldsymbol{2}}$ |  | ${}_{\boldsymbol{KW, paired}}^{\boldsymbol{2}}$ |  | ${}_{\boldsymbol{KW,paired}}^{\boldsymbol{2}}$ |
| **PR**_Ban_~**PR**_R&D_ |  | -2.9^***^ |  | -2.6^**^ |  | - |  | -1.9 |
| **PR**_Ban_~**PR**_Import_ |  | 0.03 |  | -6.6^***^ |  | - |  | -4.6^***^ |
| **PR**_Ban_~**PR**_Full_ |  | -5.5^***^ |  | -6.3^***^ |  | - |  | -4.2^***^ |
| **PR**_R&D_~**PR**_Import_ |  | 2.98^***^ |  | -4.3^***^ |  | - |  | -2.3 |
| **PR**_R&D_~**PR**_Full_ |  | -2.5^**^ |  | -3.99^***^ |  | - |  | -2.0 |
| **PR**_Import_~**PR**_Full_ |  | -5.5^***^ |  | 0.3^*^ |  | - |  | 0.3^*^ |
| **Sample size (n)** |  | n= 141 |  | n= 252 |  | n= 61 |  | n= 74 |
| **PR Mean** |  | 10.4 |  | 9.4 |  | 8.7 |  | 8.8 |
| **^a^***Fligner-Killeen* *(FK)* test of homogeneity of variances (degree of freedom=3, alpha=0.05). The *Fligner-Killeen* checks for homogeneity of variance across groups when data is non-normally distributed or when problems related to outliers in the dataset cannot be resolved.  **^b^***Kruskal-Wallis (KW)* rank sum test (degree of freedom=3, alpha=0.05). Since the PR distributions violates the homogeneity of variances assumption in *ANOVA*, we have carried out *KW* tests to compare distribution of PR across policy scenarios and separately for each risk dimension.  **^c^**Chi square derived from post hoc pair wised comparisons *Kruskal-Wallis* tests (with Bonferroni adjusted p-values, degree of freedom=3, alpha=0.05).  Note1: ******p*<0.1; ** *p*<0.05; ****p*<0.01  Note 2: Sample size for those participants that selected Environmental dimension as the most relevant is n_Env_= 141 (n_Env, Ban_=35, n_Env, R&D_=34, n_Env, Import_=36, n_Env, Full_=36).  Note 3: Sample size for those participants that selected Health dimension as the most relevant is n_Health_= 252 (n_Health, Ban_=52, n_Health, R&D_=68, n_Health, Import_=66, n_Health, Full_=66).  Note 4: Sample size for those participants that selected socio-economic dimension as the most relevant is n_Socio_= 61 (n_Socio, Ban_=12, n_Socio, R&D_=16, n_Socio, Import_=17, n_Socio, Full_=16).  Note 5: Sample size for those participants that selected Ethical dimension as the most relevant is n_Ethic_= 74 (n_Ethic, Ban_=25, n_Ethic, R&D_=15, n_Ethic, Import_=17, n_Ethic, Full_=17). | | | | | | | | |
