# Supplementary material for: Genetically modified food and consumer risk responsibility: The effect of regulatory design and risk type on cognitive information processing
Source: PLoS One. 2021 Jun 9;16(6):e0252580. doi: 10.1371/journal.pone.0252580 (PMC8189520; doi:10.1371/journal.pone.0252580)
Supplement: S7 File — (DOCX) [file pone.0252580.s007.docx]

S7 File.

Table 1. Risk statements based on risk type and with reference to the regulatory context associated with policy scenarios (See Appendix II for details on how these statements are presented to the respondents).

| **Statement ID** | **Scenario** | **Risk Dimension** | **Statements** |
| --- | --- | --- | --- |
| S1Env1 | Banned | Environmental | An adoption of GM technology may bring benefits by notably reduced levels of Greenhouse Gas (GHG) emissions, due to a decreased tractor fuel use and help to deposit Carbone dioxide (Co2) in the soil. However, banning GM technology mean disposing the chance to exploit such benefits. Hence, further environmental degradation might be a consequence of not adopting GM technology. |
| S1Env2 | Banned | Environmental | Cultivating GM crops may help farmers to reduce pesticide use in their farming. Hence, banning the cultivation of GM crops in Sweden might lead to continued use of pesticides that are harmful to the environment. |
| S1Env3 | Banned | Environmental | GM crops that are nitrogen efficient can reduce nitrogen leaching into the soil and underground water. However, banning GM technology means missing the opportunity to exploit such benefits and hence may lead to increased application of synthetic nitrogen fertilizers that are harmful for the environment. |
| S1Hea1 | Banned | Human health | Genetic engineering has applications in enriched foods (e.g., GM rice that contain additional vitamins and minerals) which is good for health status of humans. By not utilizing GM technology, we may deprive consumers from the food nutritional enrichment that GM technology offers. |
| S1Hea2 | Banned | Human health | Genetic engineering has applications in producing foods with enhanced quality (for example is derived from GM crops with lower levels of saturated fats) which is good for our health status. Therefore, banning GM technology might slow down or make it impossible to access such food quality improvements. |
| S1Hea3 | Banned | Human health | Genetic engineering may have applications in medicinal research. Therefore, totally banning GM technology might slow down or make it impossible to access potential development of medical applications of gene technology in reducing edible vaccines and drugs. |
| S1Hea4 | Banned | Human health | Some GM crops are aimed to help human nutrient intake and digestion problems (e.g., GM potato with higher amylose content could be beneficial for diabetics and in diet food). Hence, banning GM technology mean that consumers might be restricted in realizing these benefits. |
| S1Eco1 | Banned | Socio-economic | By banning GM crop cultivation in Sweden but not elsewhere in the world, Swedish farmers will lose their competitiveness, which in turn might jeopardize their future prospects in continuing their farming. |
| S1Eco2 | Banned | Socio-economic | By banning GM crop cultivation in Sweden but not elsewhere in the world, Swedish food industries might lose the advantage of food processing enhancements which in turn can jeopardize their future market opportunities. |
| S1Eco3 | Banned | Socio-economic | Adoption of GM technology might help to produce crops that are more resistant to the severe weather conditions (like harsh winters in Sweden) which would reduce the danger of crop failure. However, banning GM technology means disposing the chance to exploit such benefits. This will affect the well-being of both farmers and consumers. |
| S1Eth1 | Banned | Ethical | By banning the development of GM technology in food production, the Swedish society might lose its ability to address global problems such as the shortage of food, especially in malnourished areas. |
| S1Eth2 | Banned | Ethical | Banning GM crop cultivation in Sweden, prevent farmers benefiting from increased farm yields, hence, it might work to decrease farmers income and welfare. |
| S1Eth3 | Banned | Ethical | By banning GM technology, Swedish consumers will have less food alternatives to choose so Swedish consumers' freedom of choice might be restricted. |
| S2Env1 | R&D | Environmental | Research on genetically modified organisms can lead to gene flow from field trials into environment (the crop crosses with wild plants or non-GM crops) with potentially adverse effects on the functioning of agro ecosystems. |
| S2Env2 | R&D | Environmental | Field trials can result in uncontrolled or unintended spreading of new genes which may cross into neighbouring plants of the same or a sexually compatible species that may lead to irreversible changes in the ecosystem. |
| S2Env3 | R&D | Environmental | Cultivating GM crops may help farmers to reduce pesticide use in their farming. Hence, banning the cultivation of GM crops in Sweden might lead to continued use of pesticides that are harmful to the environment. |
| S2Hea1 | R&D | Human Health | Research field trials can fail to prevent the introduction of the GM plants into the livestock feed and human food pathways, which may lead to adverse health effects i.e., it can trigger allergies, or it can lead to toxic reactions in the digestive tract. |
| S2Hea2 | R&D | Human health | Some GM crops are aimed to help human nutrient intake and digestion problems (e.g., GM potato with higher amylose [a different wording was used above, be consistent] content could be beneficial for diabetics and in diet food). Hence, banning GM technology mean that consumers might be restricted in realizing these benefits. |
| S2Hea3 | R&D | Human health | Genetic engineering has applications in producing foods with enhanced quality (for example is derived from GM crops with lower levels of saturated fats) which is good for our health status. Therefore, banning GM technology might slow down or make it impossible to access such food quality improvements. |
| S2Eco1 | R&D | Socio-economic | By allowing GM crop cultivation only for R&D while banning it for other purposes, Swedish farmers might lose the opportunity to increase the profitability of their farm. |
| S2Eco2 | R&D | Socio-economic | By allowing GM crop cultivation only for R&D while banning it for other purposes, food industries might lose the advantage of GM food processing enhancement and market opportunities. |
| S2Eco3 | R&D | Socio-economic | Cultivating GM crops for R&D purposes may lead to gene crosses between the GM crop and neighbour non-GM crops which may inflict losses to farmers that are not cultivationg GM crops. |
| S2Eco4 | R&D | Socio-economic | Cultivating GM crops for R&D purposes might result in a situation where the GM plant crosses with wild relatives and become weed which inflict losses to neighboring farmers. |
| S2Eth1 | R&D | Ethical | Gene technology is altering the genetic material in a way that does not occur naturally by mating and/or natural recombination and development of this type of technology (even for R&D purposes) might be interfering with nature. |
| S2Eth2 | R&D | Ethical | GM technology can contribute to increase the global production of staple foods including maize, canola, and soybeans. However, Swedish society might lose its ability to contribute to find a solution to the issue of global food security if it is not allowed to use GM technology in Sweden. |
| S2Eth3 | R&D | Ethical | Allowing GM crop cultivation only for R&D purposes while banning it for farming might restrict Swedish farmers in realizing an increase in farm yields; hence it will work to decrease farmers’ income and welfare. |
| S3Env1 | Import | Environmental | An adoption of GM technology may bring benefits notably reduced levels of Greenhouse Gas (GHG) emissions, due to a decreased tractor fuel use and help to deposit Carbone dioxide (Co2) in the soil. However, banning GM cultivation may mean disposing the chance to exploit such benefits. Hence, further environmental degradation might be a consequence of not adopting GM technology. |
| S3Env2 | Import | Environmental | Cultivating GM crops may help farmers to reduce pesticide use in their farming. Hence, importing GM food and banning domestic GM crop cultivation in Sweden might lead to continued use of pesticides that are harmful to the environment. |
| S3Env3 | Import | Environmental | Nitrogen efficient GM crop varieties can be developed with the hope to reduce nitrogen leaching into the soil and underground water. However, banning domestic cultivation of GM crops might mean missing the opportunity to exploit such benefits and hence may lead to increased application of synthetic nitrogen fertilizers which are harmful for the environment. |
| S3Hea1 | Import | Human health | People that consume GM food may develop unexpected allergic and/or toxic reactions. |
| S3Hea2 | Import | Human health | Ingesting food from GM plants or animals, expose you to problems of spreading genes from GM foods to the cells of the human body, which may adversely affect human health. |
| S3Hea3 | Import | Human health | Altered genes from microorganisms like bacteria might transfer from GM foods to human digestion that are causing diseases in humans. |
| S3Hea4 | Import | Human health | There might be uncertainties regarding the health effects of long-term consumption of GM foods. |
| S3Eco1 | Import | Socio-economic | GM food producers may potentially monopolize the GM technology market by way of patenting and licensing which restricts competition as well as make it difficult for rivals to gain access to new GM technologies on fair terms. |
| S3Eco2 | Import | Socio-economic | In the case of importing GM crops from other countries, Non-GM and organic farmers may suffer losses by unintended intermixing their products with imported GM contents. |
| S3Eco3 | Import | Socio-economic | Importing GM foods might damage the reputation of organic producers since it might be hard for the agro-food industry to guarantee GM-free shipments and distribution anymore. |
| S3Eth1 | Import | Ethical | Importing GM food may pose unknown risks for consumers; hence it will have adverse effects on the general wellbeing of the population. |
| S3Eth2 | Import | Ethical | Companies producing GM food may monopolize the GM technology market by way of patenting and licensing which results in restricting competition as well as make it difficult for rivals to gain access to new GM technologies on fair terms. Hence, importing such patented products threaten the welfare of others. |
| S3Eth3 | Import | Ethical | Gene technology is altering the genetic material in a way that does not occur naturally by mating and/or natural recombination. Hence, application of this technology may mean interfering with nature. |
| S4Env1 | Full commercialisation | Environmental | Cultivation of GM crops may have a negative impact on the environment through transferring modified gene into native plant species and other agricultural crops. |
| S4Env2 | Full commercialisation | Environmental | There might be some deficiencies in the current GM food regulatory framework to evaluate sufficiently the potential Biosafety and environmental impacts of GM technology. Therefore, current regulatory frameworks may fail to detect potential adverse effects of cultivating GM crops on the environment. |
| S4Env3 | Full commercialisation | Environmental | Pesticide resistant GM crops can protect themselves against unwanted insects, but there is a risk that this modified trait has unintended effects on non-target (neutral or even beneficial) species. |
| S4Env4 | Full commercialisation | Environmental | GM plants can out-compete native species or cross with them, which potentially threatens biodiversity. |
| S4Hea1 | Full commercialisation | Human health | There might be uncertainties regarding the health effects of long-term consumption of GM foods. |
| S4Hea2 | Full commercialisation | Human health | There may be a potential danger of gene transfer from consumed GM foods to human body which may adversely affect human health. |
| S4Hea3 | Full commercialisation | Human health | There might be some deficiencies in the current GM food regulatory framework to evaluate adequately the potential health impacts of GM technology. Therefore, current regulatory frameworks may fail to detect potential adverse effects of consumption GM crops on the human health. |
| S4Eco1 | Full commercialisation | Socio-economic | Farmers that are not growing GM foods are concerned about how to preserve the identity of non-GM producer, since they have doubts about agricultural industry ability to accurately segregate GM from non-GM crops. So allowing GM food cultivation may damage non-GM food business. |
| S4Eco2 | Full commercialisation | Socio-economic | Biotechnology companies may monopolize the GM technology market by way of patenting and licensing which restrict competition as well as make it difficult for rivals to gain access to new GM technologies on fair terms. |
| S4Eco3 | Full commercialisation | Socio-economic | GM seed fees and contractual issues put farmers at a disadvantage by restricting a potential economic benefit associated with GM crops. For instance, farmers are not allowed to use the harvested seed from their own GM crop fields to be cultivated in the next period and they have to pay to seed companies. This means an increase in the cost of food production. |
| S4Eco4 | Full commercialisation | Socio-economic | Growing GM crops eventually may lead to a reduced number of varieties and natural traits in crops (since they outcompete with natural varieties) which means we will have less consumer choice in the long-term. |
| S4Eth1 | Full commercialisation | Ethical | Gene technology when applied to food may pose unknown risks for consumers as well as the environment; hence the technology has adverse effects on the general well-being of the population. |
| S4Eth2 | Full commercialisation | Ethical | Gene technology when applied to food is altering the genetic material in a way that does not occur naturally by mating and/or natural recombination. Hence, application of this type of technology may mean interfering with nature. |
| S4Eth3 | Full commercialisation | Ethical | Biotechnology companies potentially monopolize the GM technology market by way of patenting and licensing which restrict competition as well as make it difficult for rivals to gain access to new GM technologies on fair terms. |
